# HSP90 inhibitors stimulate DNAJB4 protein expression through a mechanism involving *N*^6^-methyladenosine

**DOI:** 10.1038/s41467-019-11552-8

**Published:** 2019-08-09

**Authors:** Weili Miao, Lin Li, Yonghui Zhao, Xiaoxia Dai, Xuemei Chen, Yinsheng Wang

**Affiliations:** 1 Department of Chemistry, University of California Riverside, Riverside, CA 92521-0403 USA; 2 Department of Botany and Plant Sciences, University of California Riverside, Riverside, CA 92521-0403 USA; 3 Guangdong Provincial Key Laboratory for Plant Epigenetics, College of Life Sciences and Oceanography, Shenzhen University, 518060 Shenzhen, Guangdong China

**Keywords:** Stress signalling, Chaperones, RNA modification, Mechanism of action

## Abstract

Small-molecule inhibitors for the 90-kDa heat shock protein (HSP90) have been extensively exploited in preclinical studies for the therapeutic interventions of human diseases accompanied with proteotoxic stress. By using an unbiased quantitative proteomic method, we uncover that treatment with three HSP90 inhibitors results in elevated expression of a large number of heat shock proteins. We also demonstrate that the HSP90 inhibitor-mediated increase in expression of DNAJB4 protein occurs partly through an epitranscriptomic mechanism, and is substantially modulated by the writer, eraser, and reader proteins of *N*^6^-methyladenosine (m^6^A). Furthermore, exposure to ganetespib leads to elevated modification levels at m^6^A motif sites in the 5′-UTR of DNAJB4 mRNA, and the methylation at adenosine 114 site in the 5′-UTR promotes the translation of the reporter gene mRNA. This m^6^A-mediated mechanism is also at play upon heat shock treatment. Cumulatively, we unveil that HSP90 inhibitors stimulate the translation of DNAJB4 through an epitranscriptomic mechanism.

## Abstract (web summary)

Cells respond to heat shock with transcriptional and translational adaptations but how HSP90 inhibition alters the heat shock proteome is largely unclear. Here, the authors analyze proteome changes upon HSP90 inhibition and show that an m^6^A-mediated mechanism contributes to the heat shock-induced upregulation of DNAJB4.

## Introduction

RNA is known to harbor more than 100 types of covalent modifications^1^, and the biological functions for most of these modifications remain poorly understood. Recent studies documented the widespread occurrence of *N*^6^-methyladenosine (m^6^A) in mRNA and the discovery of cellular proteins that are involved in the deposition^2–4^, recognition^5–7^, and removal^8–10^ of this modified nucleoside in mRNA. Thus, reversible methylations in mRNA may constitute a very important mechanism of gene regulation through modulating the stability and translation efficiency of mRNA^6,7,11^. In addition, this mechanism may also assume important roles in stress response^12,13^.

Heat shock response is among the best studied cellular stress response pathways, where heat shock proteins enable homeostasis of the proteome by preventing the aggregation and maintaining the native structures of proteins in cells^14^. In this vein, the 90-kDa heat shock protein (HSP90) is involved in assisting the folding of a large number of so-called client proteins^15^. Moreover, small-molecule inhibitors of HSP90 have been exploited in preclinical stage for treating various human diseases^16^. It, however, remains largely unexplored how treatment with these inhibitors modulates the heat shock proteome in human cells.

In the present study, we uncover that treatment of human cells with HSP90 inhibitors leads to substantial reprogramming of the heat shock proteome, where pronounced increases in expression are observed for DNAJB4 and HSPA1. We also find that the elevated expression of DNAJB4 occurs, in part, through an m^6^A-mediated epitranscriptomic mechanism, and a similar mechanism is at play during heat shock response.

## Results

### HSP90 inhibitors stimulate the expression of HSPs

**Fig. 1:**
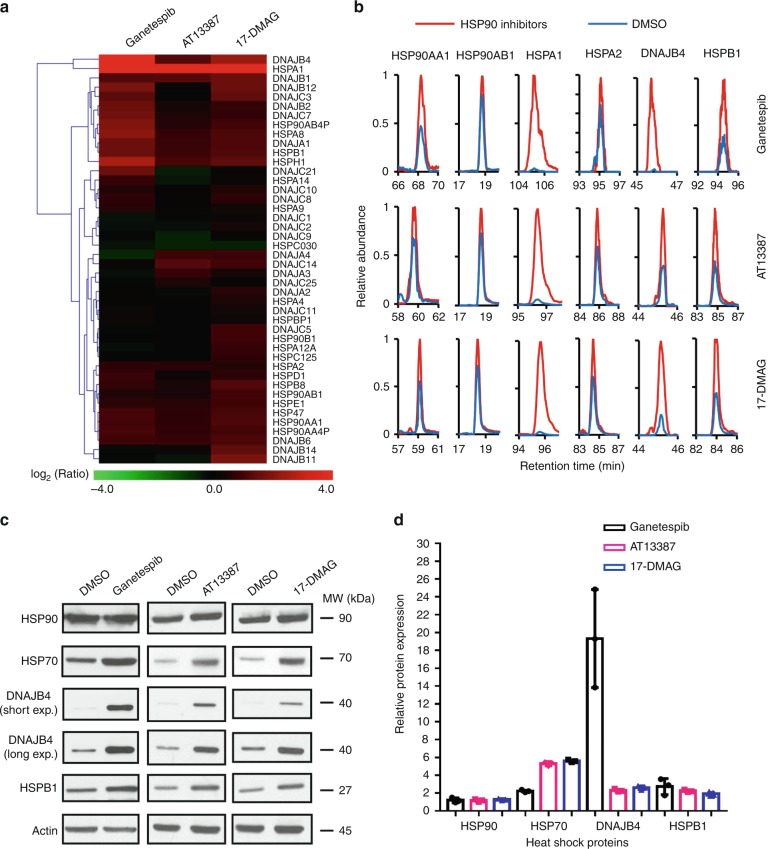
HSP90 inhibitors induced changes of heat shock proteome. **a** A heat map showing the alterations in expression levels of heat shock proteins in M14 cells upon a 24-h treatment with 100 nM of ganetespib, AT13387, or 17-DMAG. The data represent the mean of results obtained from two forward and two reverse SILAC labeling experiments (Supplementary Data 1). **b** Representative PRM traces for monitoring the relative expression levels of several heat shock proteins with or without HSP90 inhibitor treatment. **c** Western blot for validating the expression levels of select heat shock proteins after treatment with the three HSP90 inhibitors. **d** Quantification data for the differences in expression levels of heat shock proteins in M14 cells with or without HSP90 inhibitor treatment, as obtained from western blot. The quantification data represent the mean ± S.D. of results from three independent experiments. Source data are provided as a Source Data file

By employing a recently developed targeted quantitative proteomic method (Supplementary Fig. 1)^17^, we examined the alterations in expression levels of heat shock proteins in response to treatment with three small-molecule inhibitors of HSP90. These include ganetespib and AT13387 (a.k.a. onalespib) of the resorcinol chemotype, and 17-(dimethylaminoethylamino)-17-demethoxygeldanamycin (a.k.a. alvespimycin) of the ansamycin chemotype^16^. All three inhibitors interact with the ATP-binding pocket located in the N-terminal domain of HSP90^16^. Strikingly, our results showed that a number of heat shock proteins, particularly HSPA1 and DNAJB4, which belong to the HSP70 and HSP40 subfamilies, respectively, displayed markedly elevated expression in M14 melanoma cells upon treatment with the three HSP90 inhibitors (Fig. 1a, b, Supplementary Fig. 2, Supplementary Data 1).

We confirmed the altered expressions of several heat shock proteins, namely HSP90, HSP70, DNAJB4, and HSPB1, in M14 cells by western blot analysis (Fig. 1c, d). The augmented expression of HSP70 and DNAJB4 proteins was also observed in HeLa and HEK293T cells upon a 24-h treatment with ganetespib, though the magnitudes of increase were not as pronounced as what we observed for M14 cells (Supplementary Fig. 3). It is worth noting that the targeted proteomic method, which quantifies proteins on the basis of specific peptides, allows for the independent assessments of different isoforms of HSP90 (HSP90AA1 and HSP90AB1, a.k.a. HSP90α and HSP90β) and HSP70 (HSPA1, HSPA2, HSPA7 and HSPA8, a.k.a. HSP70–1, HSP70–2, HSP70B and HSC70, respectively) (Fig. 1b, d, Supplementary Data 1). The commercially available antibodies employed in our western blot analysis, however, recognize multiple isoforms of HSP90 or HSP70. Thus, the magnitudes of changes in expression of some heat shock proteins measured by western blot analysis were not as large as those determined from LC-MS/MS analysis.

**Fig. 2:**
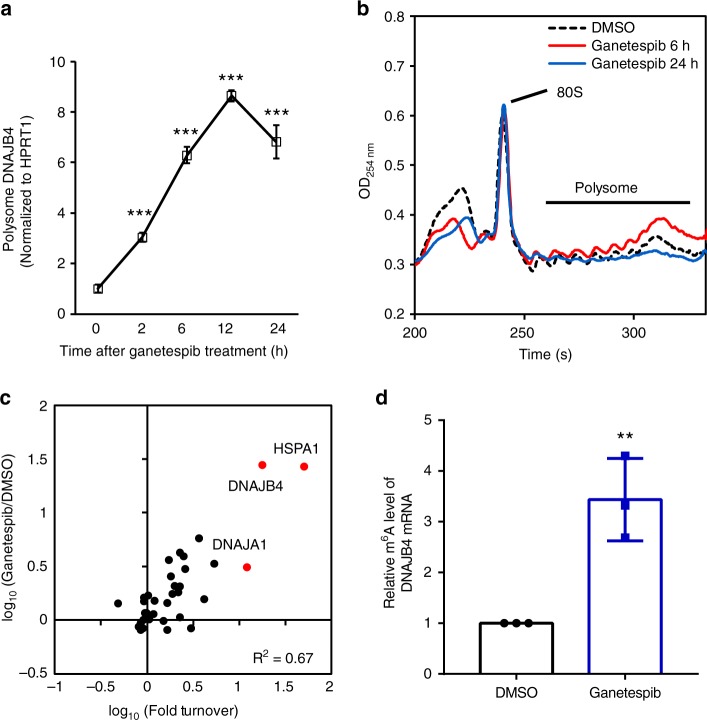
Ganetespib induced increased translation of DNAJB4. **a** RT-qPCR results show that the mRNA level of DNAJB4 exhibited a progressive increase in the polysome fraction following ganetespib treatment in M14 cells. Data were normalized to the mRNA level of *HPRT1* gene. **b** Traces for fractionation of polysome isolated from mock, DMSO-treated M14 cells or M14 cells at 6 or 24 h following treatment with 100 nM ganetespib (normalized to 80S). **c** A scatter plot shows the correlation between the expression change and protein synthesis (fold turnover) of heat shock proteins in M14 cells after ganetespib treatment. **d** m^6^A RIP-qPCR result shows the increased m^6^A level in DNAJB4 mRNA in M14 cells after a 6-h treatment with ganetespib. The data were normalized to the mRNA level of *HPRT1* gene. The quantification data represent the mean ± S.D. of results from three independent experiments. The *p-*values referred to the comparison between 0 h and the indicated time points following HSP90 inhibitor treatment and were calculated based on unpaired, two-tailed Student’s *t*-test: ^#^*p* > 0.05; *, 0.01 ≤ *p* < 0.05; **, 0.001 ≤ *p* < 0.01; ***, *p* < 0.001. Source data are provided as a Source Data file

Our quantitative proteomic data revealed a ~30-fold increase in expression level of DNAJB4 after a 24-h treatment with ganetespib (Supplementary Fig. 2); the mRNA level of this gene, however, only exhibited a 6-fold elevation (Supplementary Fig. 4a). This result suggests that ganetespib stimulates the expression of DNAJB4 protein partly through a post-transcriptional mechanism. This conclusion is further substantiated by the result obtained from polysome fractionation followed by RT-qPCR analysis, which showed augmented occupancy of *DNAJB4* mRNA in the polysome fraction after ganetespib treatment (Fig. 2a, Supplementary Fig. 4b, c). In addition, absorbance at 254 nm for the polysome fraction was increased at 6 h following ganetespib treatment, though it returned to pretreatment levels at 24-h following ganetespib treatment (Fig. 2b).

The elevated distribution of mRNA in the polysome fraction at 6-h following ganetespib treatment may be ascribed to elevated protein synthesis, which we validated for a number of heat shock proteins using a pulse-chase experiment (Supplementary Fig. 5). In particular, the results from this experiment revealed augmented syntheses of many heat shock proteins in M14 cells after a 6-h treatment with ganetespib (Supplementary Fig. 6, Supplementary Data 2). The increases in newly synthesized heat shock proteins in M14 cells upon ganetespib treatment are correlated with the elevations in their overall levels of expression (Fig. 2c).

Our proteomic data also showed that the expression levels of three other heat shock proteins (HSPA1, HSPB1, and HSPH1) were upregulated by 26-, 2.5- and 3.6-fold at 24 h following ganetespib exposure (Supplementary Data 1). Meanwhile, the mRNA expression levels of the *HSPA1, HSPB1*, and *HSPH1* genes were increased by 50-, 5- and 5-fold at the same time point (Supplementary Fig. 4a), suggesting that the HSP90 inhibitor-elicited elevation in expression levels of these three heat shock proteins occurs mainly through transcriptional regulation.

### Increased DNAJB4 expression involves an m^6^A-based mechanism

Recent studies showed that, in response to heat shock stress, cells overexpress HSP70 protein through a mechanism involving cap-independent translation enabled by m^6^A in the 5′-untranslated region (UTR) of the mRNA of *HSP70* gene^12,13,18^. Recent m^6^A sequencing studies with the use of methylated RNA immunoprecipitation (MeRIP) method revealed the presence of m^6^A peaks in multiple regions of DNAJB4 mRNA isolated from mouse embryonic fibroblast cells and HEK293T human embryonic kidney epithelial cells (Supplementary Fig. 7)^12,19^. In this context, it is worth noting that a previous residue-specific m^6^A mapping study, with the use of the m^6^A individual-nucleotide-resolution cross-linking and immunoprecipitation (miCLIP) method, only revealed two methylation sites in DNAJB4 mRNA from HEK293T cells, which are situated in the second exon and the 3′-UTR, respectively;^20^ the failure to detect m^6^A in the 5′-UTR of DNAJB4 mRNA is perhaps attributed to inadequate sensitivity of the miCLIP method. Furthermore, heat shock could result in a substantial increase in the level of m^6^A in the 5′-UTR of DNAJB4 mRNA in MEF cells (Supplementary Fig. 7a) and a nearly 4-fold elevation in the ribosomal occupancy of DNAJB4 mRNA, as revealed by m^6^A-seq and Ribo-seq analyses, respectively^12^. Therefore, we asked whether a similar mechanism contributes to the HSP90 inhibitor-induced augmentation in DNAJB4 protein.

**Fig. 3:**
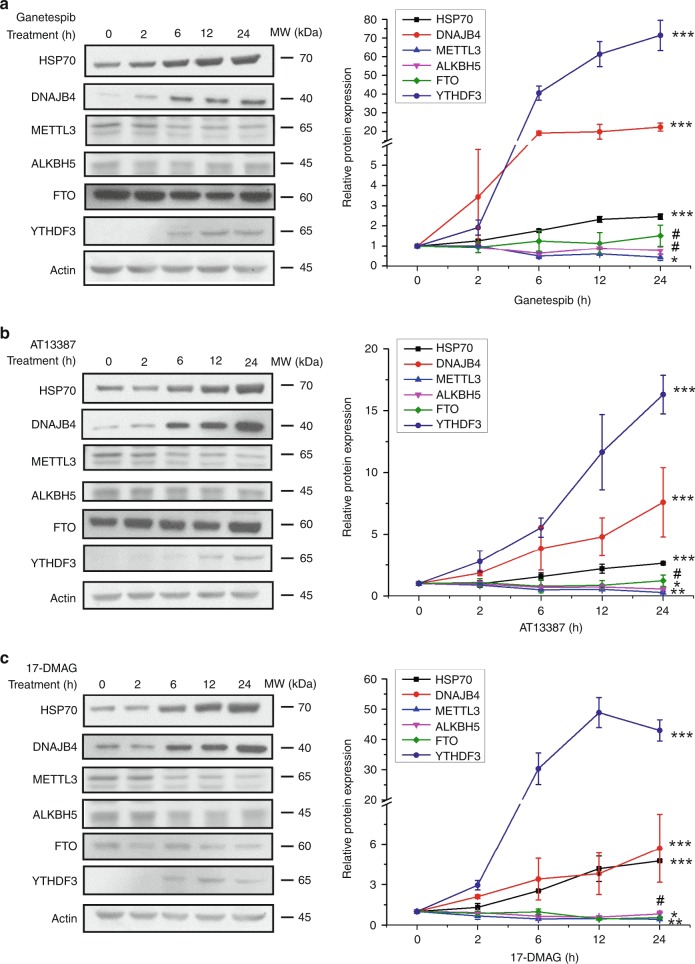
HSP90 inhibitor-induced temporal changes in protein levels. Western blot images and the quantification data showing the alterations in expression levels of the HSP70, DNAJB4, METTL3, FTO, ALKBH5, and YTHDF3 in M14 cells at different time points following treatment with 100 nM ganetespib (**a**), AT13387 (**b**), or 17-DMAG (**c**). Shown are the ratios of expression of the indicated proteins over β-actin, and further normalized to the ratios obtained for the control cells without HSP90 inhibitor treatment. The data represent the mean ± S.D. of results from three independent experiments. The *p*-values referred to the comparison between 0 h and 24 h treatment and were calculated using unpaired, two-tailed Student’s *t*-test: ^#^*p* > 0.05; *, 0.01 ≤ *p* < 0.05; **, 0.001 ≤ *p* < 0.01; ***, *p* < 0.001. Source data are provided as a Source Data file

Our m^6^A RNA immunoprecipitation (RIP) together with RT-qPCR result revealed an increased level of m^6^A in DNAJB4 mRNA after a 6-h treatment with ganetespib (Fig. 2d). We also monitored the expression levels of DNAJB4, HSP70, ALKBH5, FTO, METTL3, and YTHDF3 proteins in M14 cells at different time intervals following treatment with 100 nM ganetespib. Our results revealed a marked increase in the levels of DNAJB4 and HSP70, which paralleled the temporal profile of YTHDF3, though no substantial increase was observed for the expression level of ALKBH5, FTO, or METTL3 (Fig. 3).

**Fig. 4:**
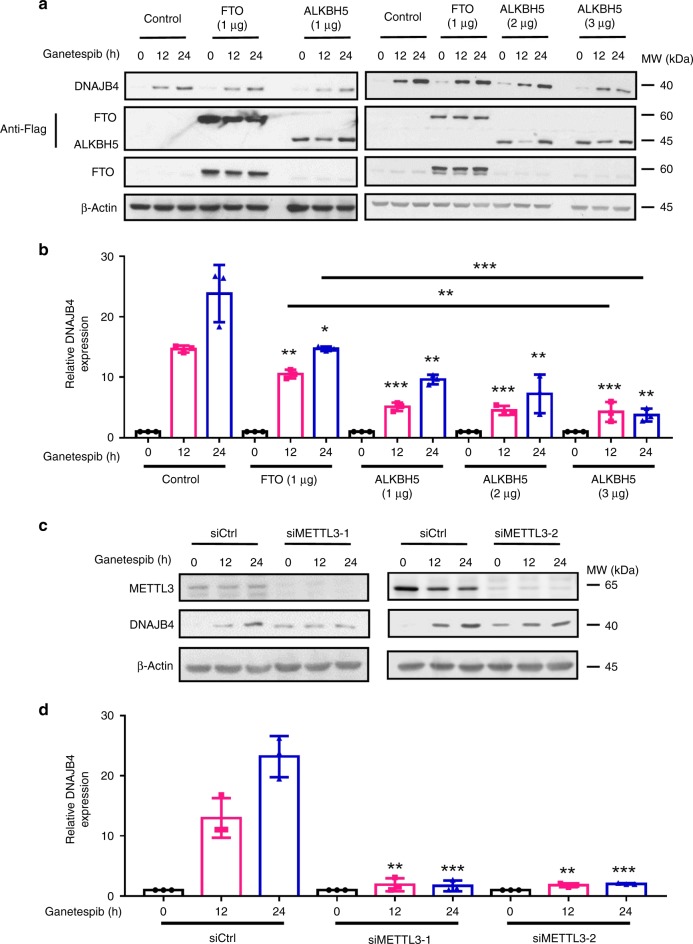
Modulation of DNAJB4 protein level by m^6^A writer and erasers. **a** Western blot for monitoring the expression levels of DNAJB4, Flag-tagged FTO, and ALKBH5, and endogenous FTO in M14 cells transfected with control plasmid or plasmids for the ectopic expression of Flag-FTO or ALKBH5 at different time intervals following treatment with 100 nM ganetespib. **b** Quantification data based on western blot analysis in (**a**). **c** Western blot for monitoring the expression levels of DNAJB4 in M14 cells treated with control non-targeting siRNA (siCtrl), siMETTL3–1 or siMETTL3–2 at different time points following treatment with 100 nM ganetespib. **d** Quantification data based on western blot analysis in (**c**). β-actin was employed as the loading control in (**a**) and (**c**). Shown in (**b**) and (**d**) are the ratios of expression of DNAJB4 protein over β-actin, and further normalized to the ratios obtained for the control cells without ganetespib treatment. The quantification data in (**b**) and (**d**) represent the mean ± S. D. of results from three independent experiments. The *p-*values referred to comparisons between control cells and cells with ectopic overexpression of the indicated genes (**b**), or between controls and siRNA-mediated knockdown of the indicated genes (**d**). The *p*-values were calculated using unpaired, two-tailed Student’s *t*-test: ^#^*p* > 0.05; *, 0.01 ≤ *p* < 0.05; **, 0.001 ≤ *p* < 0.01; ***, *p* < 0.001. Source data are provided as a Source Data file

**Fig. 5:**
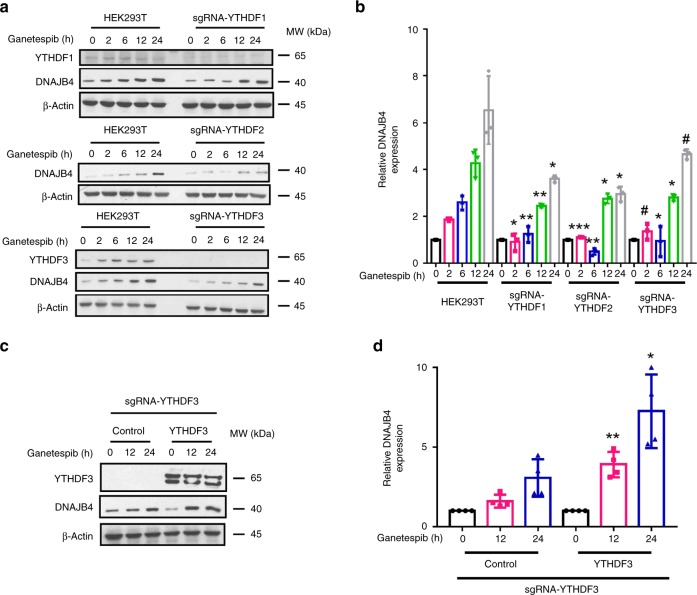
Modulation of DNAJB4 protein level by m^6^A readers. **a** Western blot for monitoring the expression levels of DNAJB4 in HEK293T cells or the isogenic cells with the *YTHDF1*, *YTHDF2*, and *YTHDF3* genes being ablated by the CRISPR-Cas9 genome editing method at different time intervals following exposure with 100 nM ganetespib. **b** Quantification data based on western blot analysis in (**a**). **c** Western blot for monitoring the expression levels of DNAJB4 in YTHDF3-deficient HEK293T cells complemented with an empty pRK7 plasmid (control) or pRK7-YTHDF3 at different time points following exposure to 100 nM ganetespib. **d** Quantification data based on western blot analysis in (**c**). β-actin was employed as the loading control in (**a**) and (**c**). Shown in (**b**) and (**d**) are the ratios of expression of DNAJB4 protein over β-actin, and further normalized to the ratios obtained for the control cells without ganetespib treatment. The quantification data in (**b**) and (**d**) represent the mean ± S.D. of results from three and four independent experiments, respectively. The *p-*values referred to comparisons between HEK293T cells and the isogenic cells with YTHDF1/2/3 genes being individually ablated (**b**), or between complementation with control and YTHDF3 plasmid (**d**). The *p-*values were calculated using unpaired, two-tailed Student’s *t*-test: ^#^*p* > 0.05; *, 0.01 ≤ *p* < 0.05; **, 0.001 ≤ *p* < 0.01; ***, *p* < 0.001. Source data are provided as a Source Data file

We next asked how the ganetespib-stimulated increase in DNAJB4 protein level is modulated by the reader, writer and eraser proteins of m^6^A. We found that ectopic overexpression of *ALKBH5* and, to a lesser degree, *FTO*, significantly attenuated the ganetespib-induced progressive increase in expression levels of DNAJB4 protein (Fig. 4a, b). In this respect, FTO was previously shown to demethylate both *N*^6^,2′-*O*-dimethyladenosine (m^6^A_m_) in the mRNA cap structure^10^ and internal m^6^A^21^, and ALKBH5 was found to demethylate internal m^6^A in mRNA^9^. The reciprocal experiment showed that small interfering RNA (siRNA)-mediated knockdown of *ALKBH5* further augmented the ganetespib-induced elevation of DNAJB4 protein (Supplementary Fig. 8). On the other hand, siRNA-mediated depletion of *METTL3*, which encodes the catalytic component of the major m^6^A methyltransferase complex^2–4^, abolished the ganetespib-induced elevated expression of DNAJB4 (Fig. 4c, d). Likewise, CRISPR-Cas9-mediated individual ablation of the three YTH domain-containing proteins (i.e., YTHDF1, YTHDF2, and YTHDF3), which are reader proteins for m^6^A^5–7^, also led to marked attenuation in the ganetespib-induced increase in expression of DNAJB4 protein (Fig. 5a, b and Supplementary Fig. 9). Furthermore, ectopic reconstitution of YTHDF3 in the YTHDF3-knockout cells rescued the elevated expression of DNAJB4 protein following ganetespib treatment (Fig. 5c, d).

We also assessed the perturbations of the heat shock proteome induced by ALKBH5 overexpression using the aforementioned targeted proteomic method. It turned out that overexpression of ALKBH5 in M14 cells only led to substantially decreased expression of DNAJB4, DNAJB12, and HSP47 (Supplementary Fig. 10a, Supplementary Data 3). Consistent with the quantitative proteomic data, western blot results revealed that ALKBH5 overexpression did not exert any apparent effect on the expression of HSPB1 protein (Supplementary Fig. 10b, c). These results underscore that ALKBH5 modulates only a small subset of heat shock proteins. In contrast to the observations made for DNAJB4, DNAJB12, and HSP47, the expression level of HSPA14 protein exhibits a marked increase upon overexpression of ALKBH5. An m^6^A site was previously identified in the last, non-coding exon in HSPA14 mRNA^20^; however, the function of this modification remains unclear. Further studies are needed to determine whether the modification level at this m^6^A site is modulated by ALKBH5 and how it affects HSPA14 translation.

**Fig. 6:**
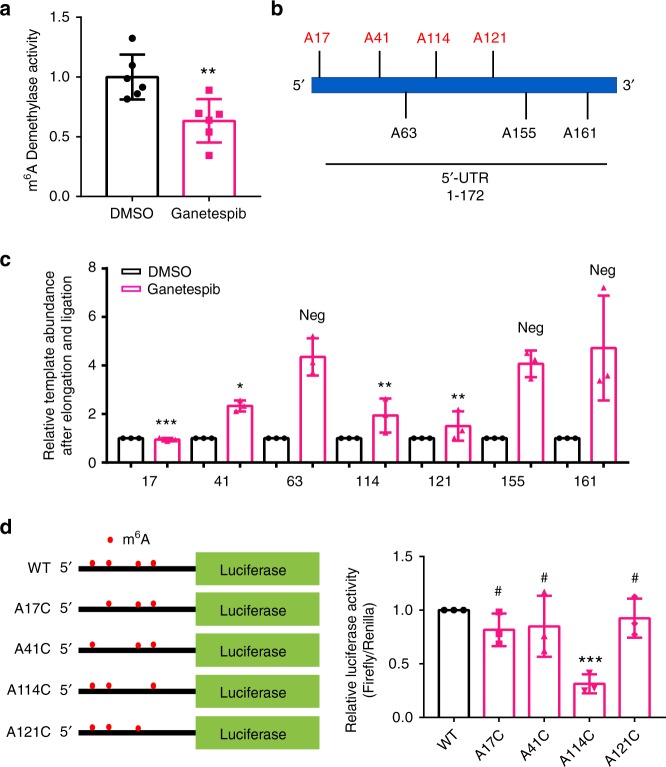
Ganetespib induces DNAJB4 translation via m^6^A in its 5′-UTR. **a** Displayed is a bar graph showing that the activity of m^6^A demethylase is decreased in M14 cells after a 6-h treatment with 100 nM ganetespib (*n* = 6). **b** A diagram showing the 5′-UTR of the human DNAJB4 mRNA and the adenosine sites monitored by the SELECT assay. The adenosine sites marked in red and black are the m^6^A motif sites and the negative control sites, respectively. **c** Relative template abundances of the 5′-UTR of DNAJB4 mRNA in M14 cells that are mocked treated with DMSO or treated with ganetespib for 6 h, as measured by the SELECT method with DMSO or ganetespib treated for 6-h (*n* = 3). A total of seven sites were chosen, four from m^6^A motif site (G**A**C) and three from U**A**A or C**A**A sites (as negative control). ‘Neg’ represents negative control. The *p*-values were calculated versus the mean value of the three negative controls. **d** Relative luciferase activities for wild-type (WT) 5′-UTR of *DNAJB4* gene and the corresponding A→C mutants (*n* = 3). Firefly luciferase activity was normalized to that of Renilla luciferase and further normalized to the wild-type construct. The data represent the mean ± S.D. of results obtained from three separate experiments. The *p-*values referred to comparison with the wild-type plasmid, and were calculated using unpaired, two-tailed Student’s *t*-test: ^#^*p* > 0.05; *, 0.01 ≤ *p* < 0.05; **, 0.001 ≤ *p* < 0.01; ***, *p* < 0.001. Source data are provided as a Source Data file

We further explored the HSP90 inhibitor-elicited increase in DNAJB4 protein synthesis by monitoring the m^6^A demethylase activity in M14 cells with or without a 6-hr treatment with 100 nM ganetespib. Our results showed that the m^6^A demethylase activity in the lysate of M14 cells was decreased to ~60% of the level observed in DMSO-treated cells at 6 hr following ganetespib treatment (Fig. 6a). This result suggests that exposure to ganetespib and the ensuing proteotoxic stress may compromise the activity of m^6^A demethylase(s) (e.g., ALKBH5).

### m^6^A modification at A114 promotes the translation of DNAJB4

Viewing that m^6^A modification in the 5′-UTR was shown to promote cap-independent translation^18^, we next asked whether the m^6^A levels in the 5′-UTR of DNAJB4 mRNA were elevated upon ganetespib treatment, and if the elevation of m^6^A promotes the translation of DNAJB4. To measure the m^6^A level at specific sites, we employed a recently reported single-base elongation- and ligation-based qPCR amplification method (SELECT)^22^. The method involves the use of two DNA probes (Up and Down Probes) that are complementary to the mRNA while leaving a single nucleotide gap at the putative m^6^A site. The single-nucleotide extension of the Up Probe by *Bst* DNA polymerase and ligation of the resulting nick between the Up and Down Probes by DNA ligase are both selectively hindered by the presence of the m^6^A modification in the mRNA template. Hence, quantitative PCR analysis of the resulting ligation product allows for quantification of the extent of m^6^A modification at the site of interest. By using this method, we monitored a total of seven adenosine sites in the 5′-UTR of DNAJB4 mRNA (Fig. 6b, Supplementary Fig. 11). These included the adenosines at all four m^6^A motif sites (AAC or GAC)^20^ and three adenosines at U/CAA sites (as negative controls) in the 5′-UTR (Fig. 6b). Our results from the SELECT assay revealed that the template abundances were increased by 4–5-fold at the three negative control sites after ganetespib treatment (Fig. 6c), which is in line with the inhibitor-induced increase DNAJB4 mRNA level (*vide supra*). Much lower increases in template levels were, however, found at all four adenosines at the m^6^A motif sites (i.e. adenosine residues 17, 41, 114, and 121, Fig. 6c), which reflect increased m^6^A levels at these sites after ganetespib treatment. This result, together with the fact that the four adenosines are situated at the m^6^A motif sites, strongly suggested that ganetespib exposure led to increased m^6^A levels at these sites in the 5′-UTR of DNAJB4 mRNA. In this context, it is worth noting the inherent limitation of SELECT assay, where the method does not provide definite evidence to support that the modified nucleosides at these adenosine sites are m^6^A.

After identifying the sites with elevated m^6^A levels, we next examined the effects of methylation at the four m^6^A motif sites on the translational efficiency of DNAJB4 by using a luciferase reporter assay. Our results showed that only mutation of adenosine 114, but not any other three adenosines at the m^6^A motif sites in the 5′-UTR, to a cytidine led to pronounced diminution (by ~3-fold) in translation efficiency (Fig. 6d), suggesting that the HSP90 inhibitor-elicited increase in m^6^A level at adenosine 114 promoted the translation of DNAJB4 mRNA.

### Heat shock stress stimulates DNAJB4 protein expression

**Fig. 7:**
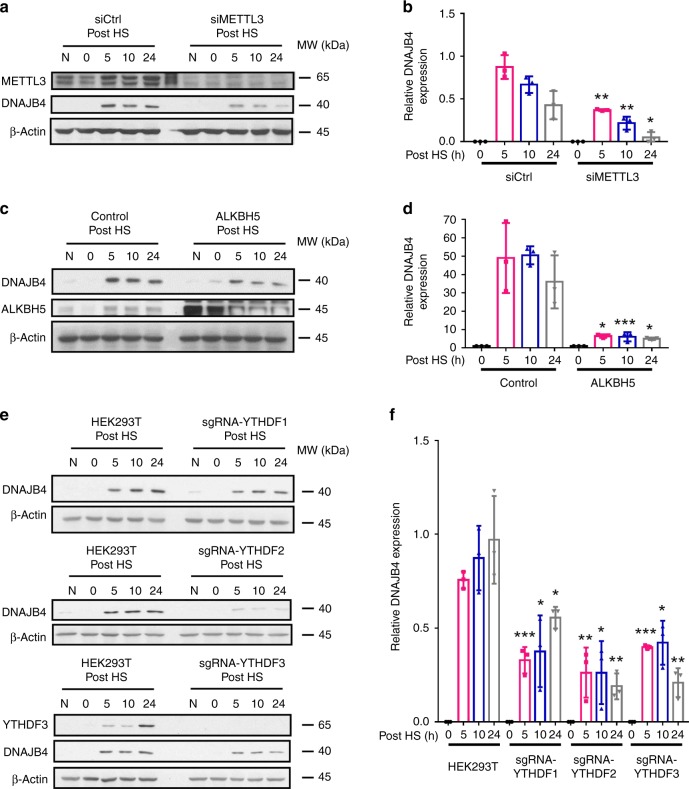
Heat shock induces elevated expression of DNAJB4 via m^6^A. Shown in (**a**), (**c**) and (**e**) are western blot images displaying the expression levels of DNAJB4 in M14 cells following heat shock treatment (HS, at 42.0 °C for 60 min), or the same cells after siRNA-mediated knockdown of METTL3 (siMETTL3–1) (**a**), ectopic overexpression of ALKBH5 (**c**), or the isogenic HEK293T cells with *YTHDF1, YTHDF2, or YTHDF3* genes being knocked out by the CRSIPR-Cas9 genomic editing method (**e**). The relevant quantification data are presented in (**b**), (**d**) and (**f**). β-actin was employed as the loading control in (**a**), (**c**) and (**e**). Shown in (**b**) and (**f**) are the ratios of expression of DNAJB4 protein over β-actin, and in (**d**) these ratios were further normalized to the ratios obtained for the cells at *t* = 0 post heat shock. The quantification data in (**b**), (**d**) and (**f**) represent the mean ± S.D. of results from three independent experiments. The *p*-values referred to the comparison between control cells and siRNA-mediated knockdown (**b**) or ectopic expression (**d**) of the indicated genes, or HEK293T cells and the isogenic cells with YTHDF1/2/3 genes being individually ablated (**f**). The *p-*values were calculated based on unpaired, two-tailed Student’s *t*-test: *, 0.01 ≤ *p* < 0.05; **, 0.001 ≤ *p* < 0.01; ***, *p* < 0.001. Source data are provided as a Source Data file

We next examined whether a similar mechanism is at work in cells under heat shock stress. It turned out that heat shock (by incubating cells at 42 °C for 1 h) again induced a marked elevation in the expression level of DNAJB4 protein, and this increase was substantially diminished in cells upon siRNA-mediated depletion of *METTL3* (Fig. 7a, b), ectopic overexpression of *ALKBH5* (Fig. 7c, d), or CRISPR-Cas9-mediated depletion of three YTH domain-containing proteins (i.e., YTHDF1–3) (Fig. 7e, f). Hence, heat shock induces the elevated expression of DNAJB4 through a similar mechanism.

## Discussion

**Fig. 8:**
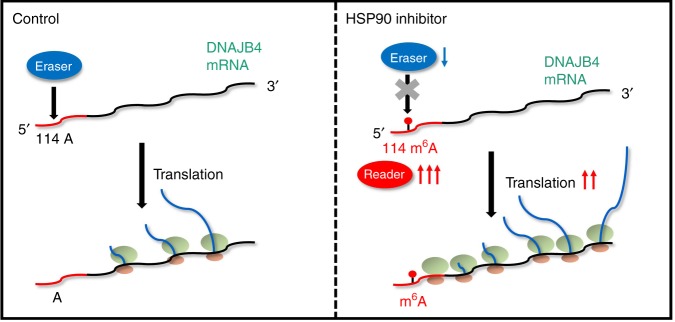
An m^6^A-based regulatory mechanism of DNAJB4 translation. Shown is a schematic diagram for the HSP90 inhibitor-induced translational upregulation of DNAJB4 through an m^6^A-mediated epitranscriptomic mechanism. 5′-UTR of DNAJB4 mRNA is marked in red and the rest of the mRNA is in black. The red dot on the DNAJB4 mRNA represents the methylation on adenosine. The green and pink circles on the DNAJB4 mRNA represent the large and small subunits of ribosome, respectively. The blue lines coming out of ribosome represent newly synthesized DNAJB4 polypeptides

Our quantitative proteomic method facilitated the assessment about the reprogramming of the heat shock proteome in cultured human cells in response to HSP90 inhibitor treatment *en masse*. We found that treatment with three HSP90 inhibitors leads to elevated expression of multiple heat shock proteins, with the most pronounced increases being observed for HSPA1 and DNAJB4. HSPA1 and DNAJB4 belong to the HSP70 and HSP40 subfamilies of heat shock proteins, where HSP40 acts as a co-chaperone for HSP70 by stimulating its ATPase activity and enhancing its interaction with substrate proteins^23^. Our study uncovers a regulatory mechanism of DNAJB4, where, in response to proteotoxic stress induced by HSP90 inhibitor treatment, cells stimulate the translation of DNAJB4 mRNA by upregulating m^6^A modification in its 5′-UTR, which is modulated by the reader, writer, and eraser proteins of m^6^A (Fig. 8). Mechanistically, we found that treatment with HSP90 inhibitor led to diminished m^6^A demethylase activity (Fig. 6a), which may arise from elevated proteotoxic stress elicited by compromised chaperone activity of HSP90. To our knowledge, there is currently no reliable method to measure independently the m^6^A methyltransferase activity in live cells or cell lysates; thus, it remains unclear whether increased m^6^A methyltransferase activity also contributes to HSP90 inhibitor-stimulated increase in m^6^A level in the 5′-UTR of DNAJB4 mRNA. Our work, together with previous studies by Zhou et al.^12,13^, showed that proteotoxic stress, arising from heat shock or treatment with HSP90 inhibitors, results in elevated translation of both HSP70 and its co-chaperone HSP40 via a common m^6^A-mediated mechanism. Thus, our work demonstrates that small-molecule HSP90 inhibitors can modulate gene expression through epitranscriptomic mechanisms.

A growing body of the literature revealed the roles of m^6^A, and its reader, writer, and eraser proteins in cancer biology^24,25^. The results from the present study suggest that the modulation of m^6^A-mediated epitranscriptomic mechanism by HSP90 inhibitors may contribute, in part, to their anti-neoplastic effects. Additionally, a previous study showed that HSP70-HSP40 chaperones inhibit the self-assembly of polyglutamine proteins into amyloid fibers^26^. In addition, HSP90 inhibitors, including 17-AAG and 17-DMAG, were found to be effective in ameliorating polyglutamine-mediated motor neuron degeneration in a mouse model of spinal and bulbar muscular atrophy (SBMA) through inducing the expression of HSP70 and HSP40 proteins^27,28^. The mechanistic underpinnings revealed from the present study suggest that the m^6^A-mediated epitranscriptomic machinery may be harnessed for treating SBMA and other neurological diseases emanating from protein aggregation and/or amyloid fiber formation.

## Methods

### Cell culture

M14 (National Cancer Institute), HeLa-S3 (ATCC, Catalog # CCL-2.2), HEK293T (ATCC, Catalog # CRL-3216), and all CRISPR-engineered cells were cultured in Dulbecco’s Modified Eagle Medium with 10% fetal bovine serum (Invitrogen, Carlsbad, CA) and penicillin (100 IU/mL). The cells were maintained at 37 °C in a humidified atmosphere containing 5% CO_2_. All cells were authenticated by ATCC with short tandem repeats (STR) profiling and tested to ensure that they were free of mycoplasma contamination with LookOut® Mycoplasma PCR Detection Kit (Sigma). The cells were treated with 100 nM HSP90 inhibitors for the indicated period of time, and, for heat shock treatment, the HEK293T cells were incubated in a 42.0 °C water bath for 60 min. We also monitored, by using a hemocytometer, the viability of M14 cells following HSP90 inhibitor treatment, where we found that a 24-h treatment with 100 nM ganetespib, AT13387 and 17-DMAG led to diminutions in the number of M14 cells by approximately 25%, 10% and 35%, respectively (Supplementary Fig. 12). For SILAC labeling experiments, M14 cells were cultured in SILAC DMEM medium containing [^13^C_6_,^15^N_2_]-lysine and [^13^C_6_]-arginine for at least 10 days to promote complete incorporation of the heavy isotope-labeled amino acids^29^. The complete heavy-isotope incorporation was confirmed by LC-MS/MS analysis (Supplementary Fig. 13). It is worth noting that heavy SILAC medium with a lower concentration of [^13^C_6_]-arginine (0.2 mM) was employed to minimize the conversion of isotopically labeled arginine to proline^30^. Under this condition, no appreciable conversion of labeled arginine to proline was observed for proline-containing peptides.

Approximately 2 × 10^7^ cells were harvested, washed with cold phosphate-buffered saline (PBS) for three times, and lysed by incubating on ice for 30 min in CelLytic M (Sigma) cell lysis reagent containing 1% protease inhibitor cocktail. The cell lysates were centrifuged at 9000 × *g* at 4 °C for 30 min, and the resultant supernatants collected.

### Plasmid and siRNAs

The sequences for siMETTL3–1 and siMETTL3–2 were 5′-CUGCAAGUAUGUUCACUAUGA-3′ and 5′-AGGAGCCAGCCAAGAAAUCAA-3′, respectively^2^. The sequences for siALKBH5–1 and siALKBH5–2 were 5′-ACAAGUACUUCUUCGGCGA-3′ and 5′-GCGCCGUCAUCAACGACUA-3′, respectively^31^. siRNA was transfected using RNAiMAX (Invitrogen) following the manufacturer’s protocol, where non-targeting siRNA (Dharmacon, D-001210–02–20) was used as control. The pcDNA3.1-DYK plasmids for ectopic expression of ALKBH5 and FTO were kindly provided by Prof. Chuan He (The University of Chicago, Chicago, IL)^32,33^. The pRK7-YTHDF3 plasmid was constructed by amplifying the cDNA of YTHDF3 from pGEX-YTHDF3 provided by Prof. Chuan He^6^ using the primers of 5′-GCTCTAGACCACCAATGTCAGATCCA-3′ and 5′-CGGGATCCTTGTTTGTTTCTATTTCTCTCCCTA-3′. The plasmids were transfected into cells by using Lipofectamine 2000 (Life Technologies), following the manufacturer’s recommended procedures.

### LC-PRM analysis

To assess the differential expression of heat shock proteins in M14 cells after HSP90 inhibitor treatment, we conducted two forward and two reverse SILAC labeling experiments, where lysates of light-labeled, inhibitor-treated M14 cells and heavy-labeled, mock (DMSO)-treated M14 cells were combined at 1:1 ratio (by mass, based on Bradford assay) in the forward labeling experiment (Supplementary Fig. 1a). The reverse labeling experiment was conducted in the opposite way. LC-MS/MS analysis in the data-dependent acquisition mode on a Q Exactive Plus hybrid quadrupole-Orbitrap mass spectrometer (Thermo Scientific, CA) showed that the ratios of total ion intensities for the light- over heavy-labeled peptides fell in the range of 0.91–1.09, where Maxquant, Version 1.5.2.8^34^, was employed to identify the light and heavy-labeled peptides and determine their intensity ratios (Supplementary Table 1). This result suggests that the equi-mass mixing of light and heavy SILAC lysates based on Bradford assay was reasonably accurate.

The proteins in the resulting light/heavy lysate mixtures were digested by trypsin using the previously reported filter-aided sample preparation (FASP) protocol^35^. Approximately 50 µg of cell lysates were washed with 8 M urea for protein denaturation using a Microcon centrifugal filter with a molecular weight cutoff of 30 kDa, and the urea buffer was then removed by centrifugation at 10,000 × *g*. The ensuing denatured proteins were reduced, alkylated, and digested with modified MS-grade trypsin (Pierce) at an enzyme/substrate ratio of 1:100 in 50 mM NH_4_HCO_3_ (pH 8.5) at 37 °C overnight. The peptide mixture was subsequently dried in a Speed-vac, desalted with OMIX C18 pipette tips (Agilent Technologies). Around 500 ng peptides were analyzed by LC-MS/MS on a Q Exactive Plus quadruple-Orbitrap mass spectrometer (Thermo Fisher Scientific) in the PRM mode. The mass spectrometer was coupled with an EASY-nLC 1200 system (Thermo Scientific), and the samples were automatically loaded onto a 4-cm trapping column (150 µm i.d.) packed with ReproSil-Pur 120 C18-AQ resin (5 µm in particle size and 120 Å in pore size, Dr. Maisch GmbH HPLC) at a flow rate of 3 µL/min. The trapping column was coupled to a 20-cm fused silica analytical column (PicoTip Emitter, New Objective, 75 µm i.d.) packed with ReproSil-Pur 120 C18-AQ resin (3 µm in particle size and 120 Å in pore size, Dr. Maisch GmbH HPLC). The peptides were then resolved using a 140-min linear gradient of 9–38% acetonitrile in 0.1% formic acid and at a flow rate of 300 nL/min. The spray voltage was 1.8 kV. Precursor ions were sequentially isolated, at a width of 1.0 *m/z*, and collisionally activated in the HCD cell at a collision energy of 29 to yield MS/MS, which were acquired in the Orbitrap analyzer at a resolution of 17500 with an AGC target of 1 × 10^5^^17^. Up to the five unique peptides with the highest precursor ion intensities for each heat shock protein were selected in the isolation list for LC-MS/MS or LC-PRM analysis, where the fragmentations of both the light- and heavy-isotope labeled peptides were monitored.

### Database search

Maxquant, Version 1.5.2.8, was used to analyze the LC-MS and MS/MS data for protein identification^34^. The database we used for the search was human IPI database, version 3.68, which contained 87,061 protein entries. The maximum number of miss-cleavages for trypsin was two per peptide. Cysteine carbamidomethylation was set as a fixed modification. Methionine oxidation and serine, threonine and tyrosine phosphorylation were set as variable modifications. The tolerances in mass accuracy were 20 ppm for MS and MS/MS acquired on the Q Exactive Plus. The maximum false discovery rates (FDRs) were set at 0.01 at both peptide and protein levels, and the minimum required peptide length was 6 amino acids.

### PRM data processing

All raw files were processed using Skyline (version 3.5)^36^ for the generation of extracted-ion chromatograms and peak integration. We imposed a mass accuracy of within 20 ppm for fragment ions during the identification of peptides in the Skyline platform. The targeted peptides were manually checked to ensure that the transitions for multiple fragment ions derived from light and heavy forms of the same peptide exhibit the same elution time in the pre-selected retention time window and display similar distribution as those in the MS/MS acquired from shotgun proteomic analysis, with dot product value being >0.7^37^. The sum of peak areas from all transitions of light or heavy forms of peptides was used for quantification.

### Pulse-chase SILAC labeling and LC-PRM analysis

M14 cells were cultured in SILAC light or heavy DMEM with 10% (v/v) dialyzed FBS, as described above. Cells cultured in heavy and light SILAC media were rinsed with PBS, and switched to light and heavy DMEM media, respectively. Immediately after the exchange of culture media, the cells were treated with 100 nM ganetespib in DMSO or mocked treated (with DMSO). After culturing for 6 h, the cells were harvested and lysates prepared for LC-PRM analysis, as described above (Supplementary Fig. 5). The fold difference in protein turnover was calculated by comparing the relative abundance of the converted proteins with or without ganetespib treatment^38^. Peptides with trypsin mis-cleavages were excluded from analysis.

### CRISPR/Cas9-mediated genome editing of HEK293T cells

CRISPR targeting was conducted following the previously reported protocols^39^, where the single guide RNAs (sgRNAs) were designed as described (http://www.broadinstitute.org/rnai/public/analysis-tools/sgrna-design)^40,41^. The guide sequences were GTGGTGAGGTATGGAATCGGAGG for YTHDF1, TGAACCTTACTTGAGTCCACAGG for YTHDF2, and ATAAAACACAACATGAATATTGG for YTHDF3, where the last three letters indicate the PAM motif. Oligodeoxyribonucleotides corresponding to target sequences were obtained from Integrated DNA Technologies and ligated into the hSpCas9 plasmid pX330 (Addgene). The constructed plasmids were then transfected into HEK293T cells using Lipofectamine 2000 (Invitrogen) in a 6-well plate and individual cells were cultured for further analysis. Genomic DNA was extracted from individual clonal cell lines, and specific DNA regions surrounding the targeted sites were screened by PCR, followed by agarose gel electrophoresis to assess the modification efficiency and by Sanger sequencing to identify the deletion loci (Supplementary Fig. 9). A set of clones with both alleles being cleaved by Cas9 were isolated, and the successful deletion of YTHDF1 and YTHDF3 was further validated by western blot analysis (Fig. 5a).

### Western blot

Cells were cultured in a 6-well plate and lysed at 60–80% confluency following the above-described procedures. The concentrations of proteins in the resulting lysates were determined using Bradford Assay (Bio-Rad). The whole-cell lysate for each sample (10 μg) was denatured by boiling in Laemmli loading buffer and subjected to SDS-PAGE separation. The proteins were subsequently transferred to a nitrocellulose membrane at 4 °C overnight. The resulting membrane was blocked with PBS-T (PBS with 0.1% Tween 20) containing 5% milk (Bio-Rad) at 4 °C for 6 h. The membrane was subsequently incubated with primary antibody at 4 °C overnight and then with secondary antibody at room temperature for 1 h. After thorough washing with PBS-T, the HRP signals were detected with Pierce ECL Western Blotting Substrate (Thermo). The uncropped western blot images are presented in the Source Data file.

Antibodies recognizing human ALKBH5 (Proteintech, 16837–1-AP, 1:50000 dilution), DNAJB4 (Santa Cruz Biotechnology, sc-100711, 1:4000 dilution), FTO (Santa Cruz Biotechnology, sc-271713, 1:2000 dilution), HSP70 (Stressgen SPA-810, 1:10,000 dilution), HSP90 (Santa Cruz Biotechnology, sc-13119, 1:10,000 dilution), HSPB1 (Santa Cruz Biotechnology, sc-13132, 1:10,000 dilution), METTL3 (Proteintech, 15073–1-AP, 1:4000 dilution), YHTDF1 (Abcam, ab99080, 1:1000 dilution), YTHDF3 (Santa Cruz Biotechnology, sc-377119, 1:500 dilution), and Flag epitope tag (Cell Signaling, 2368, 1:20,000 dilution) were employed as primary antibodies. Horseradish peroxidase-conjugated anti-rabbit IgG, IRDye® 680LT Goat anti-Mouse IgG (1:10,000 dilution) were used as secondary antibodies. Membranes were also probed with anti-actin antibody (Cell Signaling #4967, 1:10,000 dilution) to confirm equal protein loading.

### Polysome profiling

Sucrose solutions were prepared in a polysome buffer (10 mM HEPES, pH 7.4, 100 mM KCl, 5 mM MgCl_2_, 100 µg/ml cycloheximide and 2% Triton X-100). Sucrose density gradients (15–60%, w/v) were freshly prepared in SW 55 ultracentrifuge tubes (Backman) using a Gradient Master (BioComp Instruments). M14 cells were pretreated with 100 µg/ml cycloheximide at 37 °C for 7 min followed by washing with ice-cold PBS containing 100 µg/ml cycloheximide. The cells were then lysed in polysome lysis buffer. Cell debris was removed by centrifugation at 18,000 × *g* for 10 min at 4 °C. OD_254_ was measured by using a Nanodrop (Thermo) and the same amount of RNA was loaded onto sucrose gradients followed by centrifugation at 237,000 × *g* for 90 min at 4 °C in an SW 55 rotor. The resulting sample was eluted at a flow rate of 0.5 ml/min through an automated fractionation system (ISCO UA-5 UV detector, ISCO Inc.) that continuously monitored OD_254_ values. Aliquots of polysome fraction were used for real-time PCR analysis.

### m^6^A RNA immunoprecipitation (RIP)

Eighty µg of total RNA isolated from M14 cells with or without ganetespib treatment was incubated with 4 μg anti-m^6^A antibody (Millipore ABE572) in 1 × IP buffer (10 mM Tris-HCl, pH 7.4, 150 mM NaCl, and 0.1% Igepal CA-630) at 4 °C for 2 h. The m^6^A-IP mixture was then incubated with Protein A beads at 4 °C on a rotating wheel for an additional 2 h. After washing for three times with the IP buffer, the bound RNA was eluted using a 50-µL elution buffer (6.7 mM *N*^6^-methyladenosine 5′-monophosphate in 1 × IP buffer), followed by ethanol precipitation. The precipitated RNA was used for reverse transcription and real-time PCR as described below.

### Real-time PCR

M14 cells were seeded in 6-well plates at 50% confluence level. Total RNA and polysome RNA were extracted from cells or polysome fraction using TRI Reagent (Sigma). Approximately 3 μg RNA was reverse transcribed by employing M-MLV reverse transcriptase (Promega) and an oligo(dT)_18_ primer. After a 60-min incubation at 42 °C, the reverse transcriptase was deactivated by heating at 75 °C for 5 min. Quantitative real-time PCR was performed using iQ SYBR Green Supermix kit (Bio-Rad) on a Bio-Rad iCycler system (Bio-Rad), and the running conditions were at 95 °C for 3 min and 45 cycles at 95 °C for 15 s, 55 °C for 30 s, and 72 °C for 45 s. The comparative cycle threshold (Ct) method (ΔΔCt) was used for the relative quantification of gene expression^42^, and the primers are listed in Supplementary Table 2. The total mRNA level of each gene was normalized to that of the internal control (*GAPDH* or *HPRT1*). The polysome occupancy of DNAJB4 mRNA was normalized to that of internal control, *HPRT1*^6^ or *GAPDH*. The m^6^A level in the m^6^A RIP experiment was normalized to that of an internal control, *HPRT1*.

### In vitro m^6^A demethylase activity assay

The demethylase activity was measured using an ELISA-based m^6^A demethylase assay kit (ab233489, Abcam, Cambridge, MA). The M14 cells were cultured in a 6-well plate at 60–80% confluency and lysed in a buffer containing 0.7% CHAPS, 50 mM HEPES (pH 7.4), 0.5 mM EDTA, 100 mM NaCl, and 1% protease inhibitor cocktail. The demethylase activity was measured by monitoring absorbance at 450 nm using a Synergy H1 microplate reader (Biotek, Winooski, VT) and calculated following the vendor’s recommended procedures.

### Single-base elongation and ligation-based qPCR amplification

Single-base elongation and ligation-based qPCR amplification (SELECT) experiments were performed following previously reported procedures^22^. Total RNA was mixed with 40 nM Up Primer, 40 nM Down Primer (Supplementary Table 3, Gene: NM_007034.5) and 5 μM dNTP in a 17-μl 1 × CutSmart buffer. The RNA and primers were annealed by incubating the mixture using a temperature gradient: 90 °C for 1 min, 80 °C for 1 min, 70 °C for 1 min, 60 °C for 1 min, 50 °C for 1 min, and then 40 °C for 6 min. To the mixture were subsequently added a 3-μl solution containing 0.01 U *Bst* 2.0 DNA polymerase, 0.5 U SplintR ligase and 10 nmol ATP to make the final volume 20 μl. The final reaction mixture was incubated at 40 °C for 20 min, denatured at 80 °C for 20 min and subsequently cooled to 4 °C. Real-time quantitative PCR was subsequently performed using iQ SYBR Green Supermix kit (Bio-Rad) on a Bio-Rad iCycler system (Bio-Rad). The qPCR reaction (20 μl) was comprised of iQ SYBR Green Supermix, 200 nM qPCR forward primer, 200 nM qPCR reverse primer (Supplementary Table 3), 2 μl of the final reaction mixture and water. qPCR was run at the following conditions: 95 °C, 5 min; (95 °C, 10 s; 60 °C, 35 s) × 40 cycles; 95 °C, 15 s. The comparative cycle threshold (Ct) method (ΔΔCt) was used for the relative quantification of template abundance^42^.

### Dual-luciferase reporter assay

The 5′-UTR of *DNAJB4* gene was amplified by from a cDNA library prepared from M14 cells using the primers of 5′-CATGCCATGGAGGATTGAATACAGAGAC-3′ and 5′-CATGCCATGGTTCGAATGCCTTGAAAT-3′. cDNA was subcloned into NcoI-linearized pGL3-promoter vector (Gene: NM_007034.5). The A17C, A41C, A114C, and A121C mutated pGL3 plasmids were constructed by amplifying from pGL3-DNAJB4–5′-UTR using the primers listed in Supplementary Table 4. All plasmids were confirmed by Sanger sequencing.

pGL3 and pRL plasmids were co-transfected into M14 cells, and the cells were treated with 100 nM ganetespib at 18 h later. After another 6 h, the cells were lysed. The activities of firefly and Renilla luciferases were measured following the vendor’s recommended procedures (Promega), where a Synergy H1 microplate reader (Biotek, Winooski, VT) was used with a gain setting of 225, an integration time of 10 s, and a delay time of 2000 ms.

### Reporting Summary

Further information on research design is available in the Nature Research Reporting Summary linked to this article.

## Supplementary information

Supplementary Information

Description of Additional Supplementary Files

Supplementary Data 1

Supplementary Data 2

Supplementary Data 3

Reporting Summary

Source Data

## Data Availability

Excel files containing the results of the LC-PRM analyses are provided as Supplementary Data 1–3. All the raw files for both LC-MS/MS experiments and LC-PRM analyses of heat shock proteins were deposited into PeptideAtlas with the identifier number of PASS01226. The uncropped western blot image for Figs. 1c, 3a, b, c, 4a, c, 5a, c, 7a, c, e and Supplementary Figs. 3a, b, 8a, 10b are provided in the Source Data file. The ratios obtained from each individual replicate for Figs. 1d, 2a, 3a, b, c, 4b, d, 5b, d, 6c, 7b, d, f and Supplementary Figs. 3a, b, 4a, 8b, 10c are also provided in the Source Data file. All other data are available from the corresponding author on reasonable request.
