# Supplementary material for: HSP90 inhibitors stimulate DNAJB4 protein expression through a mechanism involving N6-methyladenosine
Source: Nat Commun. 2019 Aug 9;10:3613. doi: 10.1038/s41467-019-11552-8 (PMC6688989; doi:10.1038/s41467-019-11552-8)
Supplement: Supplementary file 1 — Supplementary Information [file 41467_2019_11552_MOESM1_ESM.pdf]

Supplementary Information for

"HSP90 Inhibitors Stimulate DNAJB4 Protein Expression through a Mechanism Involving *N*[6]-Methyladenosine"

Weili Miao[1], Lin Li[1], Yonghui Zhao[2], Xiaoxia Dai[1], Xuemei Chen[2] and Yinsheng Wang[1, *]

[1]Department of Chemistry, and [2]Department of Botany and Plant Sciences, University of California Riverside, CA 92521-0403

To whom correspondence should be addressed: Tel.: (951)827-2700; Fax: (951)827-4713; E-mail: Yinsheng.Wang@ucr.edu

Supplementary Table 1. LC-MS and MS/MS in the data-dependent acquisition (DDA) mode for confirming the equi-mass mixing of light- and heavy-labeled lysates in SILAC experiments.

Drug	Replicate	Total Intensity (Light Peptides)	Total Intensity (Heavy Peptides)	Ratio (Light/Heavy)
Ganetespib	Forward	6.52×10^{11}	5.97×10^{11}	1.09
Ganetespib	Reverse	9.08×10^{11}	8.77×10^{11}	1.04
AT13387	Forward	1.15×10^{12}	1.12×10^{12}	1.03
AT13387	Reverse	7.39×10^{11}	6.87×10^{11}	1.08
17-DMAG	Forward	7.42×10^{11}	6.83×10^{11}	1.09
17-DMAG	Reverse	8.28×10^{11}	9.13×10^{11}	0.91

Supplementary Table 2. Sequences for RT-qPCR primers.

Gene Name	Forward Primer	Reverse Primer
DNAJB4	5′-AAGGGTTGAAAGGAGGAGCA-3′	5′-TTCAGAATCTCTACCACCACCCA-3′
HSPA1A	5′-GGAGGCGGAGAAGTACA-3′	5′-GCTGATGATGGGGTTACA-3′
HSPH1	5′-GACAGCTGTTGCTTTGAATTACGGA-3′	5′-GCTGTTCCCAGTACCTTCAA-3′
DNAJB12	5′-CTGTGAAAAGGGTCAAGCAATGT-3′	5′-TGCCAATGGCTTTGAAGGCTT-3′
HSPB1	5′-AGATCACCGGCAAGCACGAG-3′	5′-TTGGACTGCGTGGCTAGCTT-3′
HPRT1	5′- TGACACTGGCAAAACAATGCA-3′	5′-GGTCCTTTTCACCAGCAAGCT-3′
GAPDH	5′-CCATGGAGAAGGCTGGGG-3′	5′-CAAAGTTGTCATGGATGACC-3′

Supplementary Table 3. Primer sequences for SELECT method.

Description	Primer
A17 - Up Primer	5′-tagccagtaccgtagtgcgtgTGTCTTAAGGCAGCAAGCAGACAGCG-3′
A17 - Down Primer	5′-phos/CTCTGTATTCAATCCTcagaggctgagtcgctgcat-3′
A41 - Up Primer	5′-tagccagtaccgtagtgcgtgATCAGCAATTCAGCTAGCTG-3′
A41 - Down Primer	5′-phos/CTTAAGGCAGCAAGCAGACAGCGTCTCcagaggctgagtcgctgcat-3′
A63 - Up Primer	5′-tagccagtaccgtagtgcgtgAAGCTGGGTATTTTAAAAGT-3′
A63 - Down Primer	5′-phos/AATCAGCAATTCAGCTAGCTGTCTTAAcagaggctgagtcgctgcat-3′
A114 - Up Primer	5′-tagccagtaccgtagtgcgtgAAAGAAAACAGCGTCCCCAG-3′
A114 - Down Primer	5′-phos/CTTAGCAACAGATTCTAAGAAAAATAAcagaggctgagtcgctgcat-3′
A121 - Up Primer	5′-tagccagtaccgtagtgcgtgCTTTGTAAAAGAAAACAGCG-3′
A121 - Down Primer	5′-phos/CCCCAGTCTTAGCAACAGATTCTAAGAcagaggctgagtcgctgcat-3′
A155 - Up Primer	5′-tagccagtaccgtagtgcgtgCATTTCGAATGCCTTGAAAT-3′
A155 - Down Primer	5′-phos/AACTTAGATTTCCCTTTGTAAAAGAAAcagaggctgagtcgctgcat-3′
A161 - Up Primer	5′-tagccagtaccgtagtgcgtgTTTCCCCATTTCGAATGCCT-3′
A161 - Down Primer	5′-phos/GAAATTAACTTAGATTTCCCTTTGTAAcagaggctgagtcgctgcat-3′
qPCR forward primer	5′-ATGCAGCGACTCAGCCTCTG-3′
qPCR reverse primer	5′-TAGCCAGTACCGTAGTGCGTG-3′

Supplementary Table 4. Primer sequences for mutagenesis amplification from pGL3-DNAJB4-5′UTR. The C and G in bold indicate the mutation sites, where A is mutated to C.

Description	Primer
A17C - Forward	5′-GAATACAGAG**C**CGCTGTCTGCTTGCTGCCTT-3′
A17C - Reverse	5′-AAGCAGACAGCG**G**CTCTGTATTCAATCCT-3′
A41C - Forward	5′-CTGCCTTAAG**C**CAGCTAGCTGAATTGCT-3′
A41C - Reverse	5′-TCAGCTAGCTG**G**CTTAAGGCAGCAAGCA-3′
A114C - Forward	5′-TGTTGCTAAG**C**CTGGGGACGCTGTTTT-3′
A114C - Reverse	5′-CGTCCCCAG**G**CTTAGCAACAGATTCTA-3′
A121C - Forward	5′-TAAGACTGGGG**C**CGCTGTTTTCTTTTACA-3′
A121C - Reverse	5′-AGAAAACAGCG**G**CCCCAGTCTTAGCAACA-3′

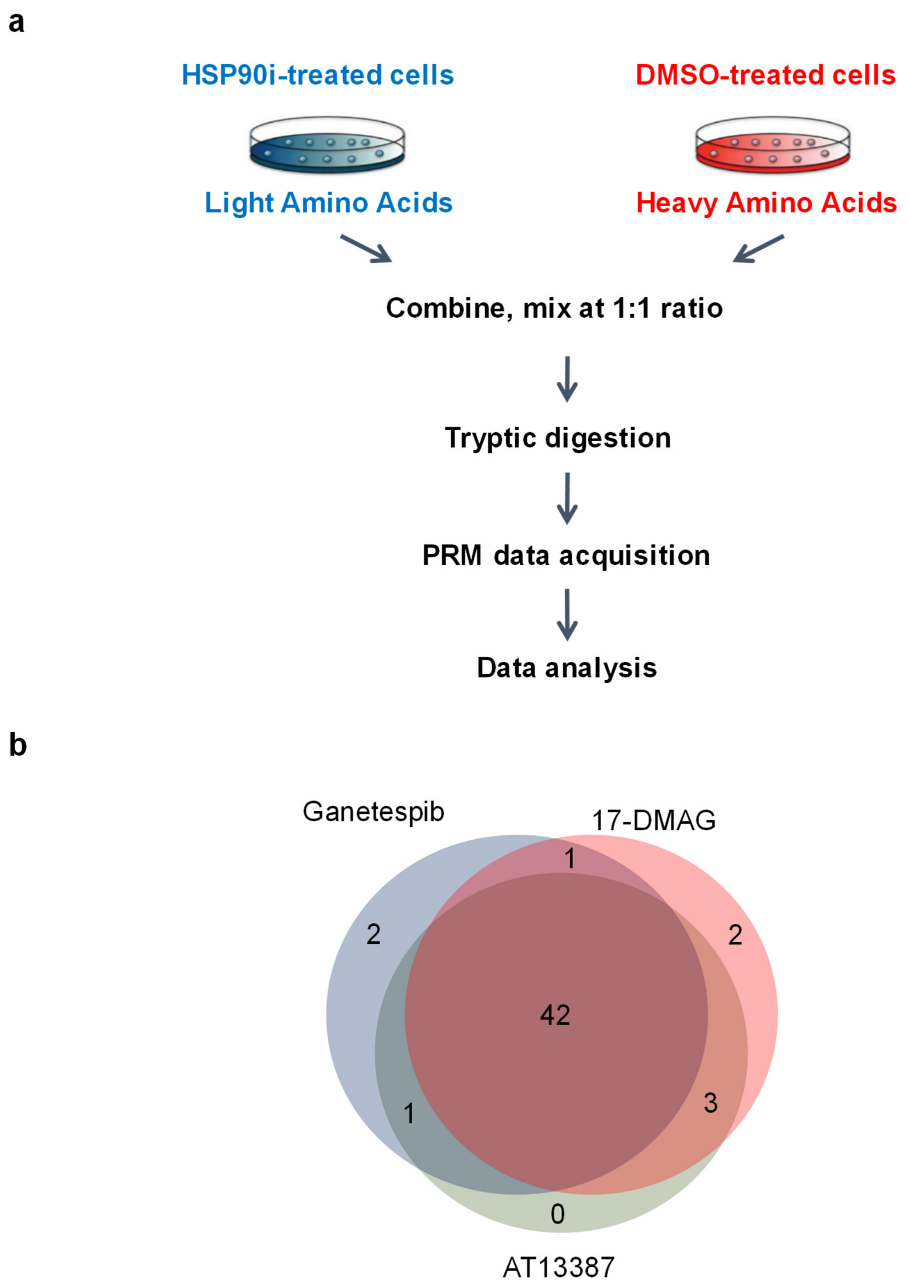

Supplementary Figure 1. SILAC coupled with LC-PRM analysis for the quantification of expression levels of heat shock proteins in M14 cells after treatment with HSP90 inhibitors (HSPi). (a) A schematic diagram showing the forward SILAC-based proteomic workflow for examining the alterations in expression levels of heat shock proteins after HSP90 inhibitor treatment. (b) A Venn diagram depicting the number of heat shock proteins quantified in M14 cells upon treatment with ganetespib, AT13387 and 17-DMAG.

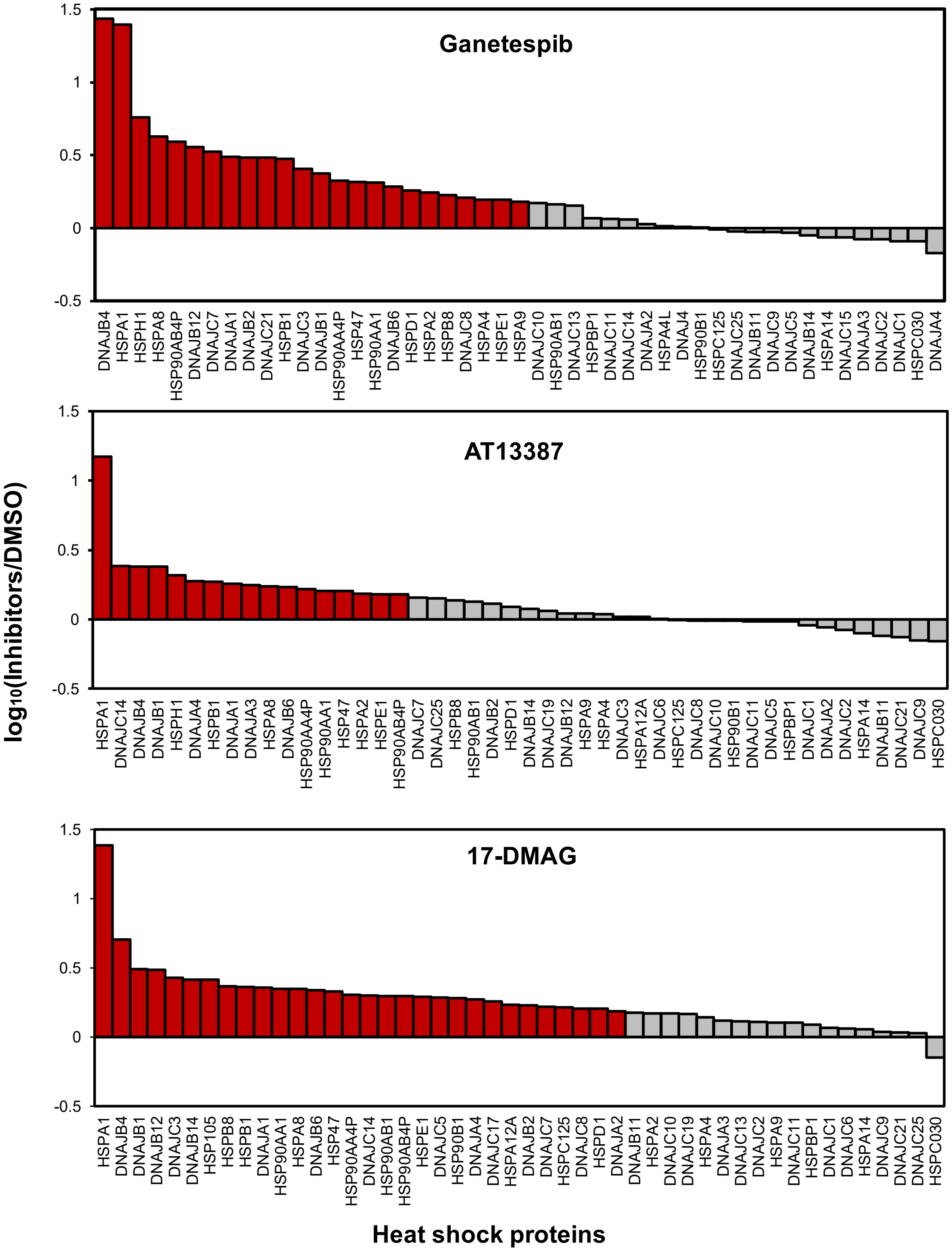

Supplementary Figure 2. Reprograming of heat shock proteome in M14 cells upon a 24-hr treatment with 100 nM of the indicated HSP90 inhibitors. Shown are $\log_{10}$(ratio) for the levels of heat shock proteins in inhibitor-treated over mock-treated (with DMSO) cells. The data represent the mean of results obtained from two forward and two reverse SILAC labeling experiments, with the ratios for the quantified peptides and proteins being listed in Supplementary Data 1. Red bars indicate those heat shock proteins that were up-regulated by at least 1.5-fold upon inhibitor treatment.

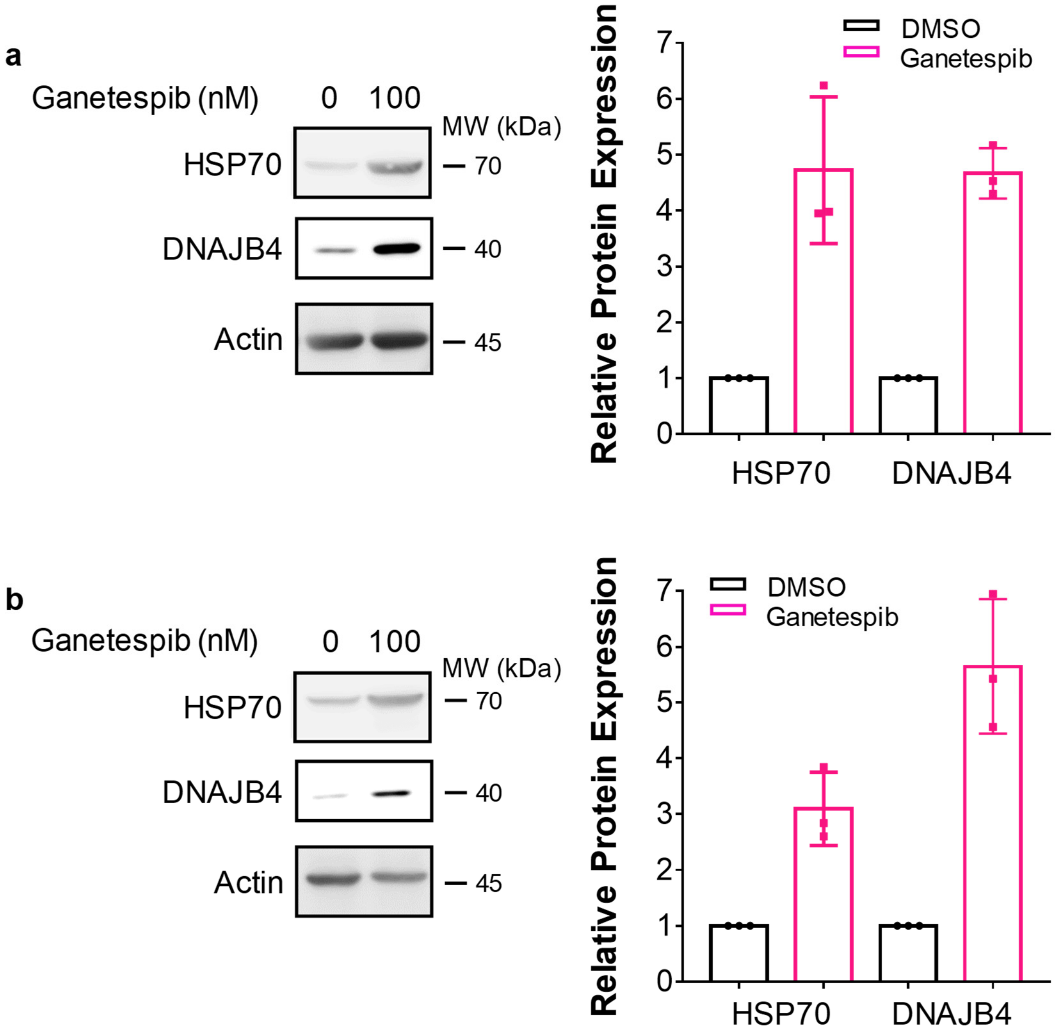

Supplementary Figure 3. Expression of heat shock proteins in HeLa and HEK293T cells after a 24-hr treatment with ganetespib. Representative Western blot images and quantification results for the expression of HSP70 and DNAJB4 proteins in HeLa (a) and HEK293T (b) cells. The quantification data in (a) and (b) represent the mean ± S. D. of results from three independent experiments. Source data are provided as a Source Data file.

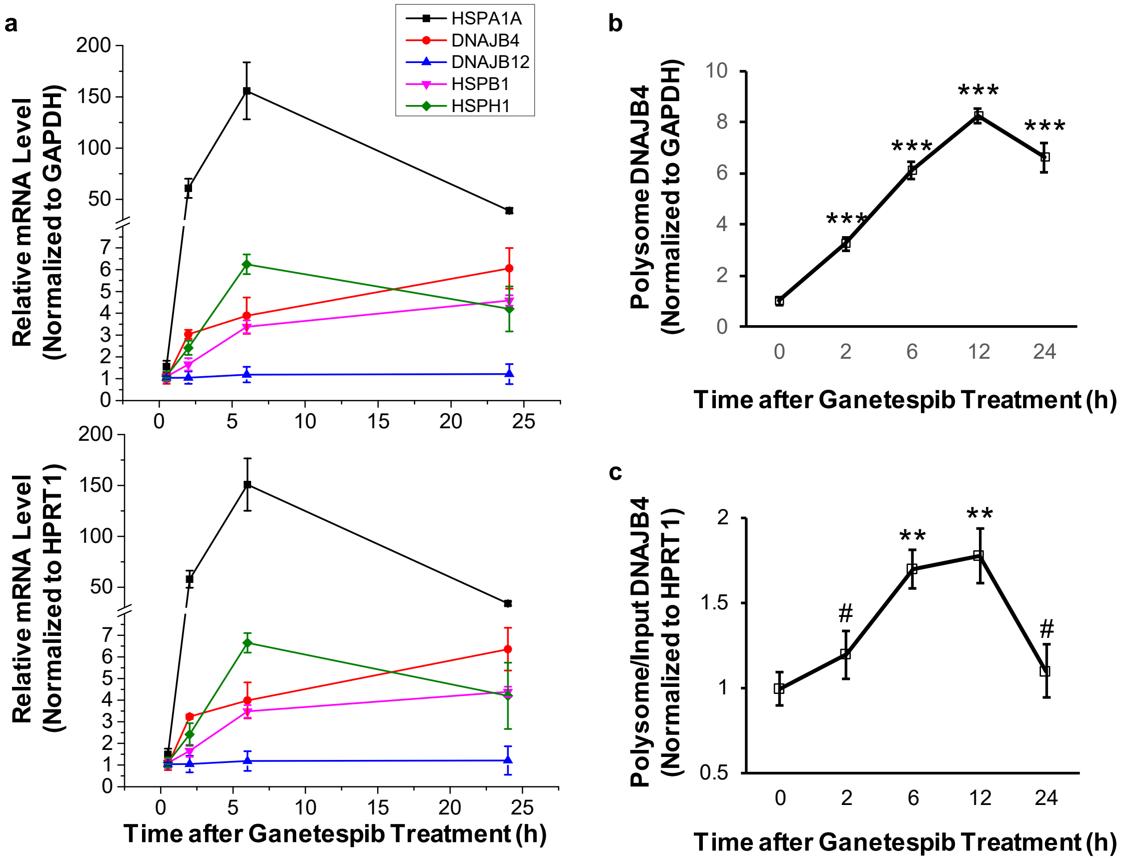

Supplementary Figure 4. RT-qPCR confirms the transcriptional and post-transcriptional mechanisms of increased expression of DNAJB4. (a) Real-time quantitative PCR for monitoring the mRNA expression levels of several genes encoding heat shock proteins in M14 cells at different time points following exposure to 100 nM ganetespib. The primers are listed in Supplementary Table 2, and *GAPDH* and *HPRT1* genes were employed as the controls. (b-c) RT-qPCR results show that the mRNA level of DNAJB4 in M14 cells exhibited a progressive increase in the polysome fraction following ganetespib treatment. Data were normalized to the mRNA level of *GAPDH* (b) or *HPRT1* (c) gene. The quantification data represent the mean ± S. D. of results from three independent experiments. The p values were calculated based on unpaired, two-tailed Student's t-test: #, $p > 0.05$; *, $0.01 \leq p < 0.05$; **, $0.001 \leq p < 0.01$; ***, $p < 0.001$. Source data are provided as a Source Data file.

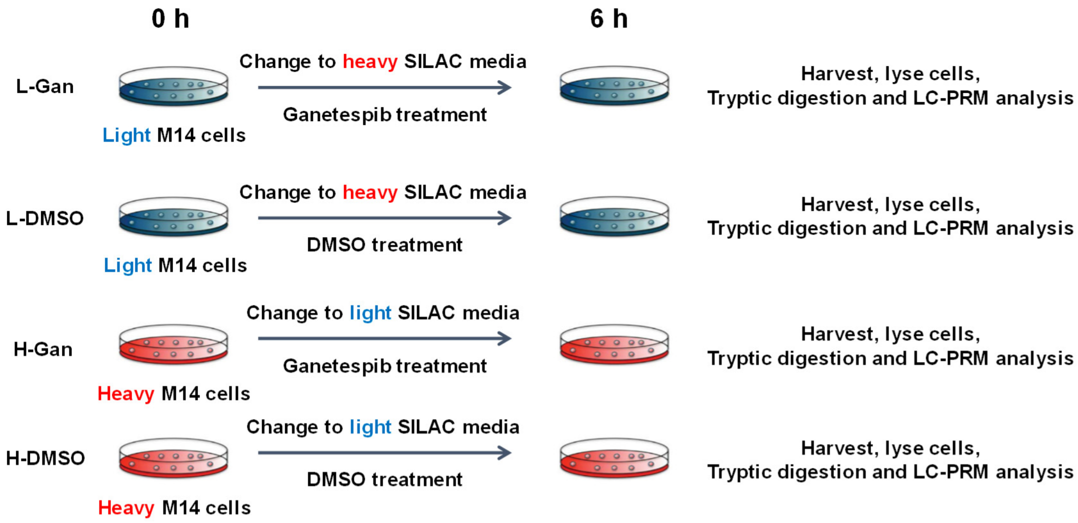

Supplementary Figure 5. Pulse-chase SILAC coupled with LC-PRM analysis for the quantification of newly synthesized heat shock proteins in M14 cells after treatment with ganetespib. A schematic diagram showing the workflow for the four pulse chase experiments during 0-6 h following ganetespib treatment.

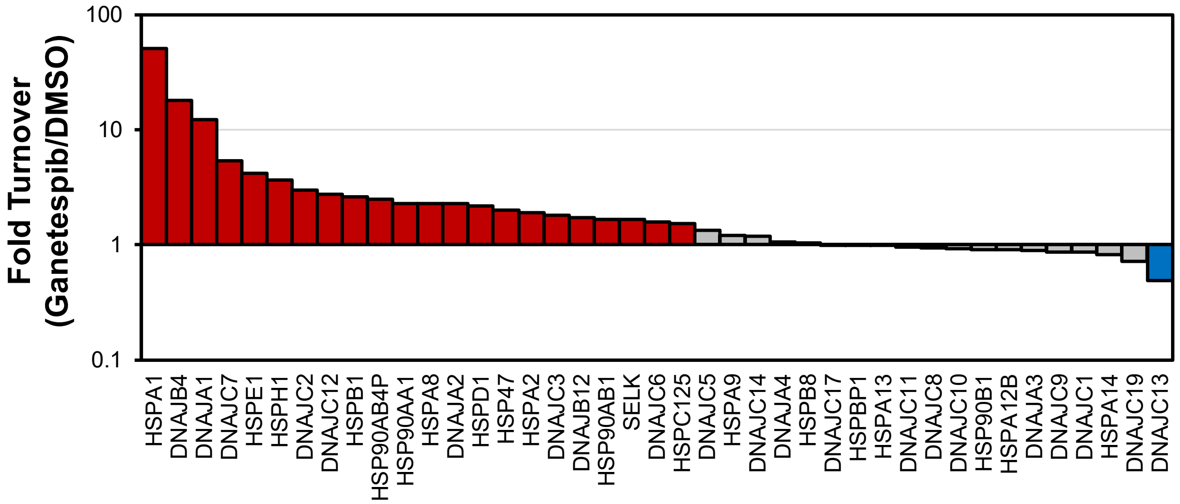

Supplementary Figure 6. Pulse-chase SILAC labeling, together with LC-PRM analysis, reveals the changes in newly synthesized heat shock proteins in M14 cells during the course of 0-6 hr following treatment with 100 nM ganetespib. A bar graph shows the fold turnover of heat shock proteins in inhibitor-treated over mock-treated (with DMSO) cells during the course of 0-6 hr following treatment with 100 nM ganetespib. The data represent the mean of results obtained from one forward and one reverse SILAC labeling experiments, with the ratios for the quantified peptides and proteins being listed in Supplementary Data 2. Red and blue bars represent those heat shock proteins that display at least a 1.5-fold increase and decrease, respectively, in protein synthesis in M14 cells upon ganetespib treatment relative to control (with DMSO treatment). The Y-axis was plotted in log_{10} scale.

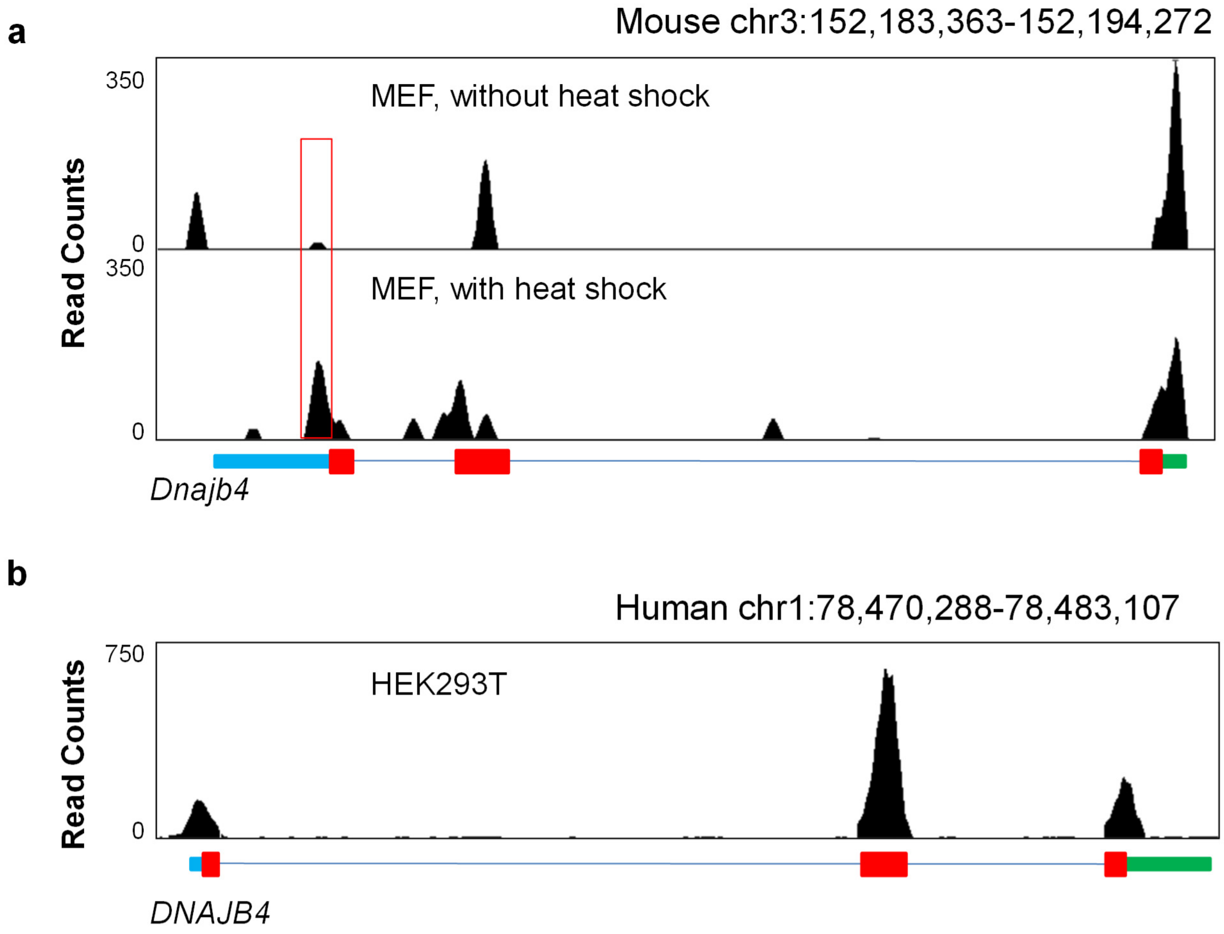

Supplementary Figure 7. Integrative genomics viewer (IGV) plots of m^{6}A peaks in mouse and human DNAJB4 mRNA. (a) IGV plots depicting m^{6}A peaks in DNAJB4 mRNA in mouse embryonic fibroblasts (MEF) with or without heat shock, as obtained by m^{6}A MeRIP. The mouse m^{6}A MeRIP data were retrieved from NCBI Sequence Read Archive under accession number SRA280261.[1] The raw data were aligned to mouse GRCm38/mm10 genome using bowtie2 2.3.4.2. The peak calling was conducted using MACS2. The data were visualized using UCSC genome browser with mouse GRCm38/mm10 assembly. The red box indicates increased level of m^{6}A in the 5′-UTR of mouse DNAJB4 mRNA. (b) IGV plot showing m^{6}A peaks in DNAJB4 mRNA isolated from HEK293T human embryonic kidney epithelial cells, as obtained by m^{6}A MeRIP. The m^{6}A MeRIP data were retrieved from the GEO database under accession number GSE29714.[2] The data were visualized using UCSC genome browser with human GRCh37/hg19 assembly. The 5′-UTR, CDS and 3′-UTR are represented by blue, red and green bars, respectively, and intron regions are indicted by thin blue line.

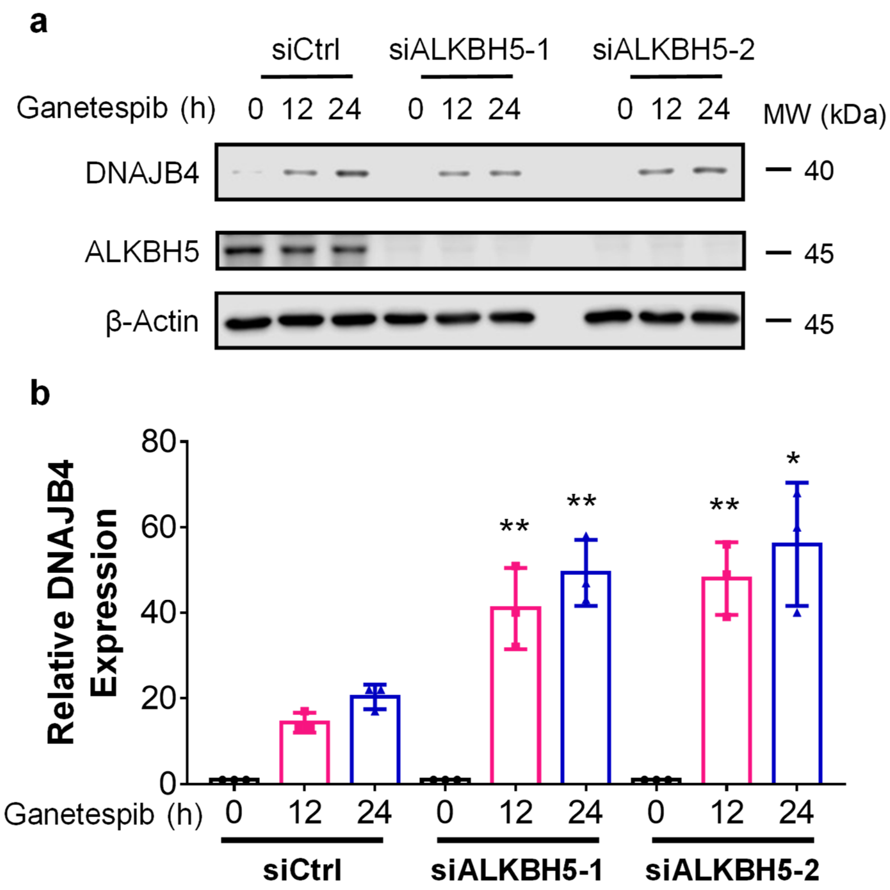

Supplementary Figure 8. The ganetespib-stimulated expression of DNAJB4 protein involves eraser protein of m[6]A, ALKBH5. (a) Western blot for monitoring the expression levels of DNAJB4 protein in M14 cells treated with control non-targeting siRNA (siCtrl), siALKBH5-1 or siALKBH5-2 at different time points following treatment with 100 nM ganetespib. (b) Quantification data based on Western blot analysis in (a). β-actin was employed as the loading control in (a). Shown in (b) is the ratios of expression of DNAJB4 protein over actin, and further normalized to the ratios obtained for the control cells without ganetespib treatment. The quantification data in (b) represent the mean ± S. D. of results from three independent experiments. The p values were calculated based on unpaired, two-tailed Student's t-test: #, $p > 0.05$; *, $0.01 \leq p < 0.05$; **, $0.001 \leq p < 0.01$. Source data are provided as a Source Data file.

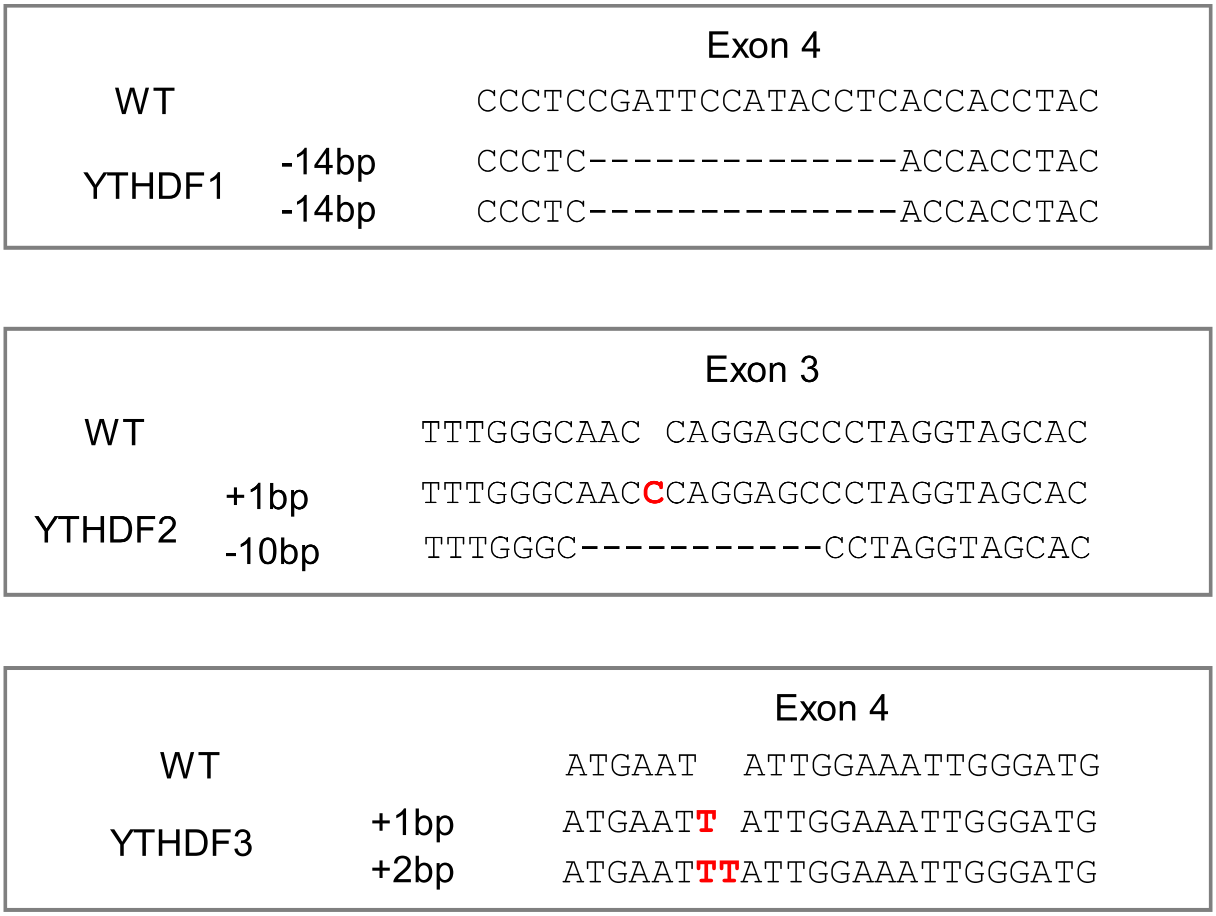

Supplementary Figure 9. CRISPR-Cas9-mediated targeting of *YTHDF1*, *YTHDF2*, and *YTHDF3* genes in HEK293T cells. Displayed are the Sanger sequencing data for confirming the out-of-frame deletions found in the three genes. The successful depletion of *YTHDF1* and *YTHDF3* were also confirmed by Western blot analysis (See Figure 5a). We were not able to validate the knockout of *YTHDF2* gene by Western blot owing to the lack of highly specific antibody.

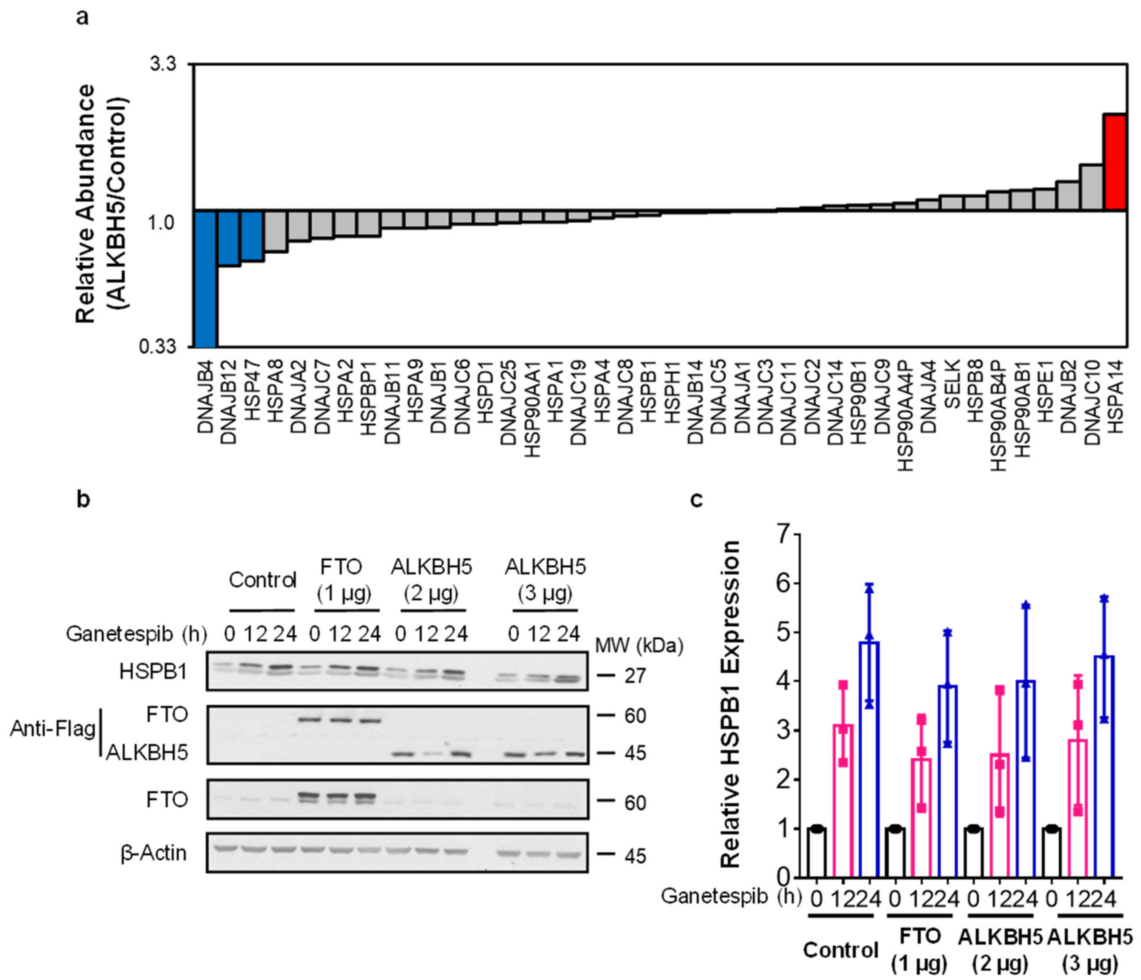

Supplementary Figure 10. ALKBH5 modulates the expression levels of some heat shock proteins during ganetespib treatment. (a) A bar graph shows the changes in expression of heat shock proteins with or without overexpression of ALKBH5 in M14 cells after a 24-hr treatment with 100 nM ganetespib. The data represent the mean of results obtained from one forward and one reverse SILAC labeling experiments, with the ratios for the quantified peptides and proteins being listed in Supplementary Data 3. Blue bars indicate those heat shock proteins that display at least a 1.5-fold decrease in protein expression in M14 cells upon ectopic overexpression of ALKBH5 (with 2 µg of ALKBH5 plasmid) relative to control. (b) Western blot for monitoring the expression levels of HSPB1, Flag-tagged FTO and ALKBH5, and endogenous FTO in M14 cells transfected with control plasmid or plasmids for the ectopic expression of Flag-FTO or ALKBH5 at different time intervals following exposure with 100 nM ganetespid. (c) Quantification data of HSPB1 based on Western blot analysis in (b). The quantification data in (c) represent the mean ± S. D. of results from three independent experiments. Source data are provided as a Source Data file.

```
1   AGGAUUGAAU ACAGAGACGC UGUCUGCUUG CUGCCUUAAG ACAGCUAGCU
51  GAAUUGCUGA UUAACUUUUA AAAUACCCAG CUUGGUUUAU UUUUCUUAGA
101 AUCUGUUGCU AAGACUGGGG ACGCUGUUUU CUUUUACAAA GGGAAAUCUA
151 AGUUAAUUUC AAGGCAUUCG AA
```

Supplementary Figure 11. The sequences of 5'-UTR (with nucleotide positions of 1-172) of the mRNA of human DNAJB4 gene (NM_007034.5). The seven adenosine sites that were monitored by the SELECT assay are highlighted in red, and all four m^{6}A motif sites (R-A*-C, where R is A or G, and A* represents the m^{6}A) in the 5'-UTR are underlined.

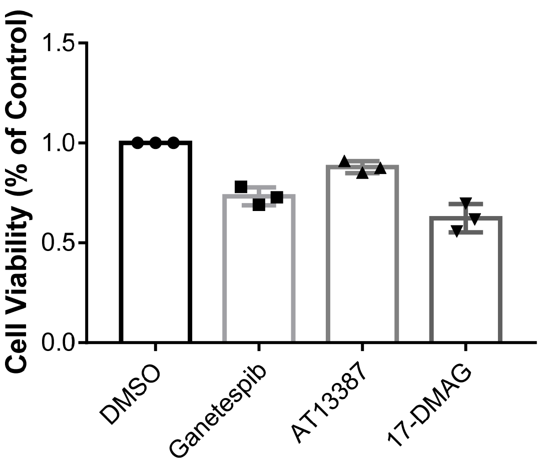

Supplementary Figure 12. The relative viability of M14 cells after a 24-hr treatment with HSP90 inhibitors (100 nM ganetespib, AT13387 or 17-DMAG).

a

	Heavy	Light
Total intensity	2.77×10^{12}	2.15×10^{10}

b

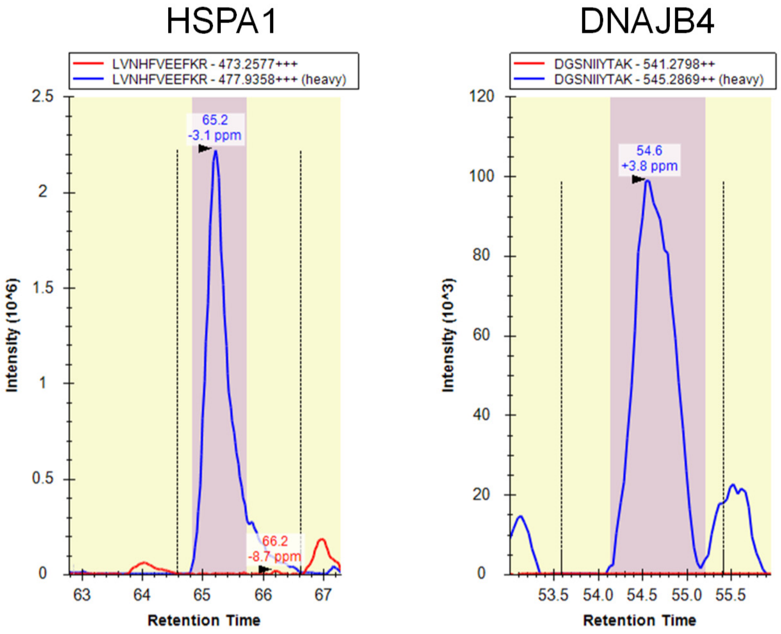

Supplementary Figure 13. LC-MS and MS/MS for the verification of complete heavy-isotope incorporation in heavy SILAC lysate. (a) The total intensities of heavy- and light-labeled peptides acquired from LC-MS/MS analysis of the tryptic digestion mixture of heavy-labeled M14 cells in the data-dependent acquisition mode. The result showed that the efficiency of heavy isotope incorporation was at least 99%. (b) PRM traces for representative tryptic peptides from HSPA1 and DNAJB4 in the tryptic digestion of heavy-labeled M14 cells.

Supplementary References:

1. Zhou, J. et al. Dynamic m^{6}A mRNA methylation directs translational control of heat shock response. *Nature* **526**, 591-4 (2015).
2. Meyer, K.D. et al. Comprehensive analysis of mRNA methylation reveals enrichment in 3' UTRs and near stop codons. *Cell* **149**, 1635-46 (2012).
