# Supplementary material for: HSP90 inhibitors stimulate DNAJB4 protein expression through a mechanism involving N6-methyladenosine
Source: Nat Commun. 2019 Aug 9;10:3613. doi: 10.1038/s41467-019-11552-8 (PMC6688989; doi:10.1038/s41467-019-11552-8)
Supplement: Supplementary file 2 — Description of Additional Supplementary Files [file 41467_2019_11552_MOESM2_ESM.docx]

**Description of Supplementary Files**

**File Name:** **Supplementary Data 1**

**Description:** Relative expression levels of heat shock proteins in M14 cells after a 24-hr treatment with 100 nM ganetespib, AT13387 or 17-DMAG, and the results were obtained from four SILAC labeling experiments (two forward and two reverse labeling) and LC-PRM analysis (in Excel).

**File Name:** **Supplementary Data 2**

**Description:** Fold turnover of heat shock proteins in M14 cells upon a 6-hr treatment with 100 nM ganetespib, and the data were obtained from pulse-chase SILAC labeling together with LC-PRM analysis. The ratios represent the fold turnover of heat shock proteins (newly synthesized protein over the protein that was originally present, in Excel).

**File Name: Supplementary Data 3**

**Description:** Relative expression levels of heat shock proteins in M14 cells with vs. without ectopic overexpression of ALKBH5 (with 2 μg plasmid) at 24-hr following ganetespib treatment, and the results were obtained from SILAC labeling and LC-PRM analysis (in Excel).
