# Supplementary material for: HSP90 inhibitors stimulate DNAJB4 protein expression through a mechanism involving N6-methyladenosine
Source: Nat Commun. 2019 Aug 9;10:3613. doi: 10.1038/s41467-019-11552-8 (PMC6688989; doi:10.1038/s41467-019-11552-8)
Supplement: Supplementary file 6 — Reporting Summary [file 41467_2019_11552_MOESM6_ESM.pdf]

nature research

Corresponding author(s): Yinsheng Wang

Last updated by author(s): July 19, 2019

Reporting Summary

Nature Research wishes to improve the reproducibility of the work that we publish. This form provides structure for consistency and transparency in reporting. For further information on Nature Research policies, see Authors & Referees and the Editorial Policy Checklist.

Statistics

For all statistical analyses, confirm that the following items are present in the figure legend, table legend, main text, or Methods section.

n/a	Confirmed	
☐	☒	The exact sample size (*n*) for each experimental group/condition, given as a discrete number and unit of measurement
☐	☒	A statement on whether measurements were taken from distinct samples or whether the same sample was measured repeatedly
☐	☒	The statistical test(s) used AND whether they are one- or two-sided<br>*Only common tests should be described solely by name; describe more complex techniques in the Methods section.*
☒	☐	A description of all covariates tested
☒	☐	A description of any assumptions or corrections, such as tests of normality and adjustment for multiple comparisons
☒	☐	A full description of the statistical parameters including central tendency (e.g. means) or other basic estimates (e.g. regression coefficient) AND variation (e.g. standard deviation) or associated estimates of uncertainty (e.g. confidence intervals)
☐	☒	For null hypothesis testing, the test statistic (e.g. *F*, *t*, *r*) with confidence intervals, effect sizes, degrees of freedom and *P* value noted<br>*Give P values as exact values whenever suitable.*
☒	☐	For Bayesian analysis, information on the choice of priors and Markov chain Monte Carlo settings
☒	☐	For hierarchical and complex designs, identification of the appropriate level for tests and full reporting of outcomes
☒	☐	Estimates of effect sizes (e.g. Cohen's *d*, Pearson's *r*), indicating how they were calculated

Our web collection on statistics for biologists contains articles on many of the points above.

Software and code

Policy information about availability of computer code

Data collection	Not applicable. No customer software or code was used for data collection or analysis
Data analysis	Not applicable. No customer software or code was used for data collection or analysis

For manuscripts utilizing custom algorithms or software that are central to the research but not yet described in published literature, software must be made available to editors/reviewers. We strongly encourage code deposition in a community repository (e.g. GitHub). See the Nature Research guidelines for submitting code & software for further information.

Data

Policy information about availability of data

All manuscripts must include a data availability statement. This statement should provide the following information, where applicable:

- Accession codes, unique identifiers, or web links for publicly available datasets
- A list of figures that have associated raw data
- A description of any restrictions on data availability

Excel files containing the results of the LC-PRM analyses are provided as Supplementary Data 1-3. All the raw files for both LC-MS/MS experiments and LC-PRM analyses of heat shock proteins were deposited into PeptideAtlas with the identifier number of PASS01226[http://www.peptideatlas.org/PASS/PASS01226]. The uncropped Western blot image for Figures 1c, 3a, 3b, 3c, 4a, 4c, 5a, 5c, 7a, 7c, 7e and Supplementary Figures 3a, 3b, 8a, 10b are provided in the Source Data file. The ratios obtained from each individual replicate for Figures 1d, 2a, 3a, 3b, 3c, 4b, 4d, 5b, 5d, 6c, 7b, 7d, 7f and Supplementary Figures 3a, 3b, 4a, 8b, 10c are also provided in the Source Data file. All other data are available from the corresponding author on reasonable request.

Field-specific reporting

Please select the one below that is the best fit for your research. If you are not sure, read the appropriate sections before making your selection.

☒ Life sciences ☐ Behavioural & social sciences ☐ Ecological, evolutionary & environmental sciences

For a reference copy of the document with all sections, see nature.com/documents/nr-reporting-summary-flat.pdf

Life sciences study design

All studies must disclose on these points even when the disclosure is negative.

Sample size	Sample sizes were described in the Methods and relevant figure legends.
Data exclusions	No data were excluded.
Replication	All experiments were performed in at least 3 biological replicates, which is indicated in the Method and Figure Legends.
Randomization	No patients or animals were used in this study.
Blinding	N/A

Reporting for specific materials, systems and methods

We require information from authors about some types of materials, experimental systems and methods used in many studies. Here, indicate whether each material, system or method listed is relevant to your study. If you are not sure if a list item applies to your research, read the appropriate section before selecting a response.

Materials & experimental systems

n/a	Involved in the study
☐	☒ Antibodies
☐	☒ Eukaryotic cell lines
☒	☐ Palaeontology
☒	☐ Animals and other organisms
☒	☐ Human research participants
☒	☐ Clinical data

Methods

n/a	Involved in the study
☒	☐ ChIP-seq
☒	☐ Flow cytometry
☒	☐ MRI-based neuroimaging

Antibodies

Antibodies used	ALKBH5 (Proteintech, 16837-1-AP, 1:50000 dilution), DNAJB4 (Santa Cruz Biotechnology, sc-100711, 1:4000 dilution), FTO (Santa Cruz Biotechnology, sc-271713, 1:2000 dilution), HSP70 (Stressgen SPA-810, 1:10000 dilution), HSP90 (Santa Cruz Biotechnology, sc-13119, 1:10000 dilution), HSPB1 (Santa Cruz Biotechnology, sc-13132, 1:10000 dilution), METTL3 (Proteintech, 15073-1-AP, 1:4000 dilution), YHTDF1 (Abcam, ab99080, 1:1000 dilution), YTHDF3 (Santa Cruz Biotechnology, SC-377119, 1:500 dilution), Flag epitope tag (Cell Signaling, 2368, 1:20000 dilution), and anti-actin antibody (Cell Signaling #4967, 1:10000 dilution). They are also described in the Source Data.
Validation	All validation statements are on the manufacturer's website. In addition, ALKBH5, METTL3, YTHDF1, and YTHDF3 antibodies were validated with siRNA KD or CRISPR KO. FTO antibody was validated with over-expression.

Eukaryotic cell lines

Policy information about cell lines

Cell line source(s)	M14 (National Cancer Institute), HeLa-S3 (ATCC, Catalog # CCL-2.2), HEK293T (ATCC, Catalog # CRL-3216)
Authentication	HEK293T and M14 cells were authenticated by ATCC on 1/21/2019 and HeLa cells were authenticated by ATCC on 3/14/2019. Cells were authenticated using using Short Tandem Repeat (STR) analysis as described in 2012 in ANSI Standard (ASN-0002) Authentication of Human Cell Lines.
Mycoplasma contamination	Not contaminated. Tested with LookOut® Mycoplasma PCR Detection Kit (Sigma).
Commonly misidentified lines (See ICLAC register)	None was used in the present study.
